# Magnitude, Temporal Trends, and Inequalities in the DALYs and YLDs of Nutritional Deficiency among Older Adults in the Western Pacific Region: Findings from the Global Burden of Disease Study 1990–2019

**DOI:** 10.3390/nu13124421

**Published:** 2021-12-10

**Authors:** Doris Y. P. Leung, Hui-Lin Cheng, Stefanos Tyrovolas, Angel S. K. Tang, Justina Y. W. Liu, Mimi M. Y. Tse, Claudia K. Y. Lai, Alex Molassiotis

**Affiliations:** 1 School of Nursing, The Hong Kong Polytechnic University, Hong Kong, China; doris.yp.leung@polyu.edu.hk (D.Y.P.L.); eileen.cheng@polyu.edu.hk (H.-L.C.); stefanos.tyrovolas@polyu.edu.hk (S.T.); sk-angel.tang@polyu.edu.hk (A.S.K.T.); justina.liu@polyu.edu.hk (J.Y.W.L.); mimi.tse@polyu.edu.hk (M.M.Y.T.); claudia.lai@polyu.edu.hk (C.K.Y.L.); 2 Parc Sanitari Sant Joan de Déu, Universitat de Barcelona, Fundació Sant Joan de Déu, 08007 Barcelona, Spain; 3 Centro de Investigación Biomédica en Red de Salud Mental, Instituto de Salud Carlos III, 28029 Madrid, Spain

**Keywords:** nutritional deficiency, malnutrition, older adults, western pacific, the global burden of disease

## Abstract

The population in the Western Pacific region is aging rapidly. Nutritional deficiency is prevalent in older adults; however, information regarding nutritional deficiency in this population is scarce. Using the 2019 Global Burden of Disease (GBD) results, the age-standardized disability-adjusted life years (DALYs) and years of healthy life lost due to disability (YLDs) from nutritional deficiency were estimated between 1990 and 2019 for this population. Average annual percentage change (AAPC) was used to assess temporal trends, and linear mixed-effects models were used to examine socioeconomic and sex inequalities. From 1990 to 2019, the age-standardized DALYs of nutritional deficiency in this population decreased from 697.95 to 290.95 per 100,000, and their age-standardized YLDs decreased from 459.03 to 195.65 per 100,000, with the greatest declines seen in South Korea (AAPCs < −5.0). Tonga had the least decline in DALYs (AAPC = −0.8), whereas Fiji experienced an increase in YLDs (AAPC = 0.1). Being female and having a lower sociodemographic index score was significantly associated with higher age-standardized DALYs and YLDs. The magnitude and temporal trends of the nutritional deficiency burden among older adults varied across countries and sex in the region, indicating that health policies on nutritional deficiency among older adults must be crafted to local conditions.

## 1. Introduction

The Western Pacific region has one of the largest aging populations in the world, accounting for 34.3% of older adults in the world aged 65 years or over [1]. The size of this older population is projected to double over the next 30 years, from 240 million to 480 million by 2050 [1,2]. Within this aging population, it is estimated that the number of oldest-old adults (aged ≥80 years) will triple during this period [2]. Some countries in the region, such as Singapore, Vietnam, the Lao People’s Democratic Republic, and China, are expected to transition from an aging to an aged society in less than 30 years [1]. Aging populations are prone to chronic diseases, functional decline, and cognitive impairment, placing a significant burden on regional healthcare resources [3].

Nutritional deficiencies have been identified as an important risk factor in the disease burden of older adults through increasing rates of infections, lifestyle diseases, frailty, sarcopenia, and mental disorders [4,5]. This is prevalent in older adults, manifesting in deficiencies in energy, protein, and other micronutrients including vitamins (e.g., vitamin D) and minerals (e.g., iron), thereby leading to the progression of under-nutrition [4,6,7]. Owing to physiological and psychosocial factors, many older adults have diminished dietary intake and poor food options, both of which would result in a reduction in the quantity and quality of the nutrients that they consume [7,8]. Meta-analytic results indicate that, with regard to prevalence, the risk of high malnutrition ranges from 8.5% in a community setting, to 28.0% in a hospital setting [9,10]. Studies have shown that nutritional deficiencies are related to an increased risk of morbidity and mortality [11,12,13], and thus are a serious public health problem that has been attracting more attention from policymakers [14].

Recent research in the area has extended from older patients in hospitals, care situations and nursing homes, to community settings [15,16]. However, the scope of inquiry has remained limited to the district level, as well as to health resource outcomes. A recent study has estimated the disease burden due to the nutritional status of 186 countries from 1990 to 2015, using the Global Nutritional Index calculated based on disability-adjusted life years (DALYs). DALYS associated with nutritional deficiency are defined as the sum of the number of years of healthy life lost, due to the premature loss of life (YLL) and to disability (YLD) in the population from cases of nutritional deficiency [17]. That study clearly showed the impact of socioeconomic factors on the nutritional burden. There was substantial variation among low- and middle-income countries in the Western Pacific region, compared with other WHO regional groups in 2015 [18]. That study provided information about temporal trends in the disease burden owing to nutritional deficiencies at the country level. However, this revealed little about demographic metrics, such as age-specific estimates for older adults and gender inequality, with regard to the health burden of the disorder, data needed by nutritionists, public health professionals, and policy makers.

Nutritional experts in the Western Pacific region have acknowledged the lack of country-specific data on nutrition in the older adult subpopulation [19]. In this study, we aimed to fill this knowledge gap by providing data on (1) the magnitude of the problem, by calculating age-standardized DALYs and YLDs and (2) temporal trends, by estimating the average annual percentage change (AAPC) in age-standardized DALYs and YLDs attributed to nutritional deficiency in countries in the region from 1990 to 2019. Lastly, (3) we examined gender inequality and socioeconomic disparities in relation to the burden of nutritional deficiency in older adults.

## 2. Materials and Methods

### 2.1. Data Source

The data utilized in this study are available on the Global Burden of Disease, Injuries and Risk Factors Study (GBD) website (http://ghdx.healthdata.org/gbd-results-tool, accessed date: 27 October 2021), which provides specific data on multiple measures of loss of health from 369 causes and 87 risk factors for 204 countries and territories from 1990 to 2019 [17]. We extracted the annual DALYs and YLDs numbers on nutritional deficiency according to sex, age (≥65 years old), and the sociodemographic index (SDI) from 1990 to 2019 for the 27 countries or territories in the Western Pacific region. These 27 countries or territories can be classified into three economic levels (high income, upper-middle, and lower-middle income) based on the World Bank Classification in 2019 [20]. In GBD, the International Classification of Diseases (ICD) 10th version codes D50-D53.9, E00-E02, E40-E46.9, E50-E61.9, E64-E64.9, Z13.2-Z13.3, and 9th version codes 244.2, 260–269.9, 280–281.2, V12.1, V18.2-V18.3, V77.2, V78.0-V78.1 were used to represent nutritional deficiency, which includes protein-energy malnutrition, and deficiencies in iron, vitamin A, iodine, and other nutritional deficiencies [21].

To quantify the trends in burden due to nutritional deficiencies in the Western Pacific region, the age-standardized rates of DALYs and YLDs, expressed per 100,000 population, were calculated for 30 consecutive years according to the following age stratification used in GBD: 65–69, 70–74, 75–79, 80–84, 85–89, 90–94, and >95 years old, using the GBD world population in 2019 as the standard population. The age-standardized rate is a weighted average of the age-specific rates per 100,000 persons, where the weights are the proportions of persons in the corresponding age groups of a standard population. Age-standardization is essential when comparing different populations with different age distributions, because it removes the influences of population size and age structure. Briefly, YLDs present the number of years lived in less than “ideal health.” DALYs are the sum of the number of years of life lost (YLLs are the years lost due to premature mortality) and of YLDs [17,22].

The SDI is used to further assess the effects of socioeconomic factors on trends in the burden of nutritional deficiency over time. The SDI is a summary index of components covering country-level income per capita, average educational attainment among individuals over the age of 15, and total fertility rate among women under the age of 25. The SDI ranges from 0 to 100, with a higher value indicating a higher level of socioeconomic development [21].

More details on the GBD methodology and estimates analysis can be found in previous GBD 2019 publications [17,22,23].

### 2.2. Statistical Analysis

Stratified by sex, the annual percent change (APC) and the average annual percentage change (AAPC) in the age-standardized rates of DALYs and YLDs attributed to nutritional deficiency over the years were computed for the Western Pacific region, and for each of the 27 countries and territories in the region using the joinpoint regression programme 4.8.0.1 [24]. The AAPC is a weighted average of the APCs, and has a 95% confidence interval (CI), which was used as a summary measure of the trend over the complete study period. The trend is considered stable if the 95% CI of the AAPC covers 0; decreasing if the upper bound of the 95% CI is <0; and increasing if the lower bound of the 95% CI is >0. The statistical significance of the AAPC was tested using the parametric t-test provided by the joinpoint regression programme.

Pooled analyses were performed to examine associations between age-standardized DALYs and YLD rates for nutritional deficiency with SDI and sex, using data from all years and countries by Spearman correlation and independent t-test with a 95% bootstrap confidence interval (CI). To interpret the strength, we classified correlations into three groups: weak (≤0.3), moderate (0.4–0.6), and strong (≥0.7). To further examine the individual contribution of sex and SDI and their interaction effect on age-standardized DALYs and YLDs rates attributed to nutritional deficiency, mixed-effect multilevel linear regression models were performed. This was performed to assess whether these two measures of burden due to nutritional deficiency were related to sex and socioeconomic development (i.e., SDI) between 1990 and 2019, after controlling for the dependency of the observations at the country level. Specifically, two linear mixed-effect model regressions were carried out separately, for age-standardized DALYs and YLD rates. In the models, four independent variables, namely, time (1990–2019), sex (male vs. female), SDI, and an interaction term of Sex × SDI, were considered as the level 1 analysis. Countries were considered for the level 2 analysis where the observations of the repeated measures were aggregated. Statistical tests on AAPC were performed using the joinpoint regression programme, although all other statistical tests were performed using SPSS version 26.0. *p*-values were calculated based on two-sided tests and the level of significance was set at 5%.

## 3. Results

### 3.1. Age-Specific Crude Rates of DALYs and YLDs Attributed to Nutritional Deficiency in the Western Pacific Region

**Figure 1:**
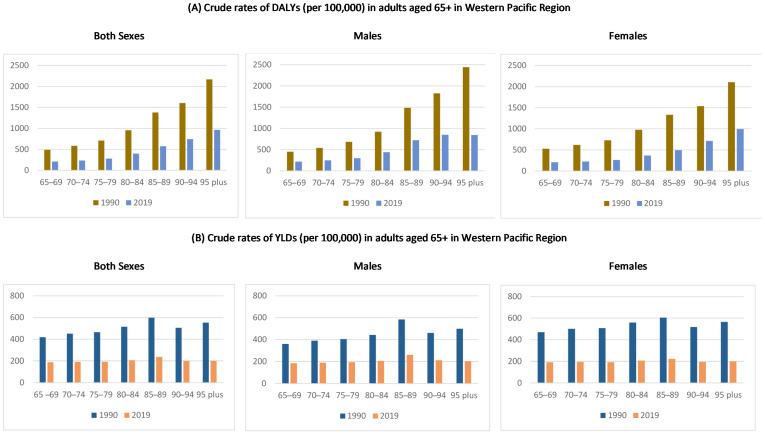
Age-specific (**A**) DALYs, (**B**) YLD crude rate by both sexes, male, and female, in the Western Pacific region from 1990 to 2019. Note: Points in the *Y*-axis indicate estimates of the rates per 100,000 population.

Age-specific crude rates of DALYs and YLDs among older adults in the Western Pacific region, stratified by sex, are shown in Figure 1 and the Supplementary Table S1. The age-specific DALYs rates were reduced from 1990 to 2019, and were found to increase with age in both sexes, reaching a maximum in the age group of 95 years and above. Comparing between the two years, a sex difference in the impact of nutritional deficiency was observed in 1990, in which the crude DALYs rates were larger in females than males; however, in 2019 the rates were comparable between males and females (Figure 1A). For YLDs, the age-specific crude rates were also reduced over the study period, and were similar in all age groups and between males and females (Figure 1B).

### 3.2. Temporal Trends in DALYs and YLD Attributed to Nutritional Deficiency in the Western Pacific Region

Table 1 shows the overall age-standardized DALYs and YLD rates and their percentage change in nutritional deficiency among adults aged 65 or above in the Western Pacific region, in the period of 1990 to 2019. In the whole region, both the age-standardized DALYs and YLDs rates decreased significantly during the period 1990–2019, with larger drops in females than males. Specifically, the estimated age-standardized DALYs rate for both sexes decreased from 697.95 (95%CI: 696.03, 699.87) in 1990 to 290.95 (95%CI: 290.25, 291.65) per 100,000 population in 2019—an annual percentage change of 3.0% (2.2%, 3.8%). The decrement in the age-standardized rate in YLDs was also observed in males by an average of 2.6% (2.5%, 2.8%) and females by an average of 3.3% (3.0%, 3.6%), respectively. For YLDs, a decreasing pattern was also observed. The age-standardized YLDs rate decreased significantly by an average of 2.9% (2.8%, 3.1%) in both sexes, by 2.5% (2.3%, 2.6%) in males, and 3.2% (3.1%, 3.3%) in females.

**Figure 2:**
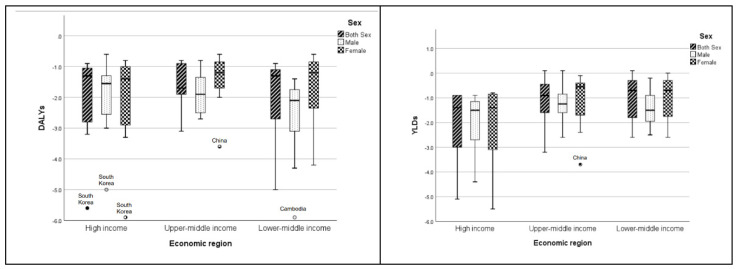
Boxplots of the average annual percentage change in age-standardized DALYs and YLDs rates attributable to nutritional deficiency among older adults (65+) from 1990 to 2019 in the Western Pacific region.

Analyses of the AAPC of age-standardized rates in DALYs and YLDs attributed to nutritional deficiency among older adults between 1990 and 2019 in both sexes, and by country in the Western Pacific region, are presented in Figure 2, Table 2, and Supplementary Figure S1. All of the countries have significant decreasing trends in age-standardized DALYs rates in both sexes over the period, as reflected by the upper bound of the 95% CI of the AAPCs being <0. However, a substantial variation was observed by country, with AAPC ≥5 in two countries (South Korea and Cambodia), between 3 and 4 in four countries (Laos, Vietnam, Singapore, and China), between 2 and 3 in one country (Brunei), and <2 in the remaining 20 countries. Larger variations were observed in the lower-middle income region (Figure 2 and Table 2). The age-standardized DALYs rate (per 100,000 population) ranged from 129.12 (95% CI: 117.32, 140.92) in New Zealand to 4920.64 (4832.42, 5008.86) in Cambodia in 1990, and from 84.32 (77.68, 90.96) in New Zealand to 3140.23 (2397.29, 3883.17) in Kiribati in 2019, in the data for both sexes. In addition, variations in the magnitude of the decreasing trends in the age-standardized rate in DALYs by sex were observed, with most countries seeing sharper reductions in males than in females. Exceptions were observed in seven high-income or upper-middle income countries (South Korea, Japan, Singapore, Brunei, Malaysia, China and Fiji), where there were sharper reductions in females. The age-standardized DALYs rates for males ranged from 106.91 (89.55, 124.27) in New Zealand to 6282.01 (6118.17, 6445.85) in Cambodia in 1990, and from 71.01 (62.05, 79.97) in New Zealand to 1725.12 (818.98, 2631.26) in Kiribati in 2019. The corresponding ranges for females were 145.07 (128.81, 161.33) in New Zealand to 5135.66 (3561.68, 6709.64) in Kiribati in 1990, and from 96.27 (86.51, 106.03) in New Zealand to 3894.84 (2879.90, 4909.78) in Kiribati in 2019. For all of the countries, the patterns of the decreasing trends varied as well. Although, in general the decreases occurred gradually over time in the region, the age-standardized rate in DALYs in six countries increased first, and then decreased. These countries were: Japan (in males only); Tonga; Nauru; Mongolia; Vanuatu (in females only), and Papua New Guinea (Supplementary Figure S1).

For the age-standardized YLDs rate, contrary to most countries that showed significant decreasing trends, Fiji and Vanuatu showed significant increasing trends in both sexes, with AAPCs = 0.1 (0.00, 0.2) (*p*-values < 0.05). Among the 25 countries with decreasing trends in the age-standardized rate in YLDs, substantial variations were observed, with AAPC ≥5 in one country (South Korea), between 3 and 4 in three countries (Japan, Singapore, and China), between 2 and 3 in three countries (Malaysia, Vietnam, and Cambodia), and below 2 in the remaining 18 countries. Larger variations were observed in high-income regions (Figure 2 and Table 2). The age-standardized YLDs rate ranged from 95.91 (85.79, 106.03) in New Zealand to 1093.73 (1034.99, 1152.47) in Laos in 1990, and from 68.53 (62.47, 74.59) in New Zealand to 853.57 (526.07, 1181.07) in Kiribati in 2019, in the data for both sexes. Similar to the age-standardized rate in DALYs, most countries saw sharper reductions in males than females, with the exception of five high-income countries (South Korea, Japan, Singapore, New Zealand, and Brunei), three upper-middle income countries (Malaysia, China, and Fiji), and one lower-middle income country (Vanuatu), with sharper reductions in females. In 1990, the highest age-standardized YLDs rate for males was 943.84 (854.42, 1033.26) in Laos, and the lowest was 69.96 (56.24, 83.68) in New Zealand, whereas in 2019 the highest rate was 545.98 (505.08, 586.88) in Laos and the lowest was 53.86 (46.02, 61.70) in New Zealand. The corresponding figures for females were as follows: the highest was 1244.06 (594.34, 1893.78) in Kiribati; the lowest was 111.89 (105.41, 118.37) in Australia in 1990; the highest was 1157.38 (689.12, 1625.64) in Kiribati and the lowest was 81.56 (72.46, 90.66) in New Zealand in 2019. Variations in the pattern of a decreasing trend in the age-standardized rate in YLDs for all of the countries are presented in Supplementary Figure S1.

### 3.3. Relationship of the Age-Standardized Rates in DALYs and YLDs with Sex, SDI, and Time

Mean values of the age-standardized rates in DALYs and YLDs attributed to nutritional deficiency and their correlations with SDI by sex are presented in Table 3. On average, females demonstrated a significantly greater age-standardized rate in DALYs (mean difference = 506.44, 95% CI (422.42, 598.28), *p*-value < 0.001) and in YLDs (mean difference = 387.76, 95% CI (360.82, 416.22), *p*-value < 0.001) than their male counterparts. Strong and negative correlations between the age-standardized DALYs rate attributed to nutritional deficiency and SDI (correlations < −0.84, *p*-values < 0.001) were observed in both sexes, males and females. For YLDs, negative correlations with SDI were observed, with strong correlations in both sexes and females (correlations < −0.84, *p*-value < 0.001) and moderate correlations (correlation = −0.682, *p*-value < 0.001) in males.

The results of the mixed-effects model regression for age-standardized rates in DALYs and YLDs on Time, Sex, SDI, and Sex × SDI as level 1 factors, and countries as a level 2 factors, are presented in Table 4. The two models showed that the age-standardized rates in DALYs and YLDs have significant negative associations with sex and SDI. In addition, the interaction of sex and SDI was also statistically significant with a coefficient of 1317.44 for DALYs and 1216.17 for YLDs, after controlling for the effect of time and the clustering effect of the country. This indicates that older adults who were males and living in countries with high SDI tended to have lower age-standardized rates in DALYs and YLDs. The impact of SDI on reducing the two measures of burden was greater among males than females.

## 4. Discussion

Regionally, our results from the Western Pacific area indicate that nutritional deficiency imposes a formidable burden on older adults. They also show that although there is a trend of a decrease in burden, DALYs and YLDs were decreasing to a small extent in most low- and middle-income areas in the region, over the study period. Socioeconomic and gender inequality persist. A higher burden attributed to nutritional deficiency was still seen in countries in the region with lower SDI, and in females. More importantly, females living in lower SDI countries in the region experienced a greater burden from nutritional deficiency.

The present study found that although there has been a substantial reduction in the Western Pacific region in the age-standardized DALYs and YLDs rates attributed to nutritional deficiency from 1990 to 2019, the magnitudes were notably larger with higher DALYs, and increased with age. Despite the significant burden of increased disability related to nutritional deficiency in this vulnerable population, government policies seldom prioritize the development of effective strategies to meet their nutritional needs. Only a few high-income countries in the region, including Australia, Singapore, and New Zealand, have drawn up nutritional guidelines or recommendations specifically for older adults [25,26,27]. Nonetheless, the challenges involved in addressing nutritional deficiency in older adults have not been emphasized. Although international dietetic organizations recommend that all older people, regardless of age, can benefit from routine assessments of nutritional risk and careful attention to nutritional adequacy and balance [4,28,29], older adults, particularly the old-old, may be at a higher risk from consuming food of poor quality, especially when the consumption of food and its access and preparation present difficulties for them, due to physical and/or cognitive decline, and/or the lack of a caregiver. Some evidence exists regarding the health benefits of nutritional strategies targeting this population in the United States and European countries, through the provision of assisted meal services or packed foods, or the mobilization of social or community resources to support the nutritional care of older adults [30,31,32]. These nutritional support strategies can be considered for adoption in the Western Pacific region, especially as populations in the region continue to age. A further analysis could focus on subtypes of nutritional deficiency to determine areas of priority in dealing with the burden, so that policies can be developed for the older adult subpopulation in the Western Pacific region.

With regard to spatial patterns in the burden of nutritional deficiency in the Western Pacific region, the age-standardized rates in DALYs and YLDs were high in lower-middle income countries, especially those with lower SDI. Our jointpoint and mixed models results are in line with those of previous studies, which found a strong relationship between dietary quality and socioeconomic status among older adults, as reflected in energy intake and macronutrient and micronutrient levels [33]. Dietary habits in the Western Pacific area have been recognized as a major health risk factor for the population [19]. However, to date the focus has been on maternal and child nutrition [34]. Research and guidelines for older populations have been lacking, although this population is expected to increase dramatically in the coming years in the Western Pacific region. Our analysis showed that the majority of countries reported a mild decline (AAPC <2) in both DALYs and YLDs, attributed to nutritional deficiencies. Countries implementing long-term nutrition policies and strategies such as South Korea, Japan, and Singapore saw large declines in DALYs or YLDs attributed to nutritional deficiencies [35,36,37]. Similar moderate reductions were noted among some Western Pacific regions (i.e., Malaysia), where population-healthy nutrition policies have been implemented for several years [38,39]. In contrast to the above, the island of Fiji and Vanuatu showed an increase in AAPCs in both sexes. Although these places have taken nutrition-related actions [40,41], the current trends call for additional efforts to be made in reforming health and nutrition, as well as in enhancing nutrition policies, especially those relating to older adults. These results, in conjunction with our analysis, show the impact of nutrition and public health policies on the health of the older adult population. Our study is the first to report the diverse geographical/regional disability patterns attributed to nutritional disorders, using population-based data from the GBD 1990–2019 study, indicating that country-specific nutrition strategies are needed in Western Pacific areas.

Our research also reveals that there has been a great difference in the past 20 years between males and females in DALYs and YLDs, attributed to nutritional deficiency. Although the age-standardized rates in DALYs and YLDs attributed to nutritional deficiency show a decreasing trend in both sex groups, substantially larger burdens were observed in women than men in most of the countries in the region, especially in the low- and upper-middle income countries, as shown in Supplementary Figure S1. The result is in line with published literature indicating that women of all ages report a greater magnitude of disability than men [42,43,44]. Apart from the differences in genetic background between men and women [45], social factors such as financial deprivation and social inequality may play a role in creating these disparities [46]. Differential power relations between sex subgroups are clear in countries with lower SDI in the Western Pacific area [47,48]. Social inequality may be apparent in women, with respect to their health circumstances and their pursuit of access to the healthcare system. This social inequality in women may further be amplified with the interplay between poverty and limited availability of health care in the community, meaning that women are unlikely to treat their health conditions in a timely manner. Hence, they might live with a disability for a longer time than men. On the other hand, the rise of female empowerment and better availability of healthcare services are expected to close the gender gap in high-income countries in the region [48]. Nevertheless, our study has provided country- and sex-specific data regarding the burden attributable to nutritional deficiency among older adults. This information is important for developing nutritional initiatives for older people in the Western Pacific region [19].

Our study has some limitations, including the use of GBD 2019 data on burden metrics being calculated from estimates based on a Bayesian hierarchy model, using pooled data obtained from an extensive systematic review at the global, regional, and national level; bias may have resulted due to statistical assumptions and the data sources in the estimations [17,22,23]. Moreover, we used the aggregate data at the country level instead of at the district level, which might have introduced bias from geographical variations within a country in DALYs and YLDs estimates. Additional limitations are that the data analyses (such as in correlations and mixed-effect modeling) could not adjust for other factors influencing nutritional deficiencies, such as living alone, following specific dietary schemes and others, that could also have altered the estimations. As this is a descriptive research work, although we applied a number of correlates and associations (i.e., Spearman correlations and mixed models), our analyses did not come to any conclusion about causal relationships.

## 5. Conclusions

Using the GBD data of 27 countries from 1990–2019 in the Western Pacific region, a diverse age-standardized DALY pattern attributable to nutritional disorders was observed. Although the results show a great difference between males and females in age-standardized DALYs and YLDs, such a gap is narrowing over time. Older adults who were males and living in high SDI countries tended to have lower age-standardized rates in DALYs and YLDs. Moreover, age-specific DALYs increased with age, reaching a maximum in the age group of 95 years and above. As an aging population would likely drive up resource utilization and healthcare costs, our findings call for governments to be highly aware of the importance of formulating nutritional policies that address the needs of older people, especially those of the female sex, to assure gender equality in health.

## Figures and Tables

**Table 1:** Trends in age-standardized DALYs and YLDs rates due to nutritional deficiency among older adults (65+) from 1990 to 2019 in the Western Pacific region.

Burden	Sex	1990	2019	AAPC
DALYs	Both sexes	697.95 (696.03, 699.87)	290.95 (290.25, 291.65)	−3.0 (−3.8, −2.2)
	Males	676.66 (673.38, 679.94)	314.45 (313.31, 315.59)	−2.6 (−2.8, −2.5)
	Females	721.59 (719.09, 724.09)	274.61 (273.71, 275.51)	−3.3 (−3.6, −3.0)
YLDs	Both sexes	459.03 (457.55, 460.51)	195.65 (195.09, 196.21)	−2.9 (−3.1, −2.8)
	Males	401.88 (399.64, 404.12)	195.56 (194.70, 196.42)	−2.5 (−2.6, −2.3)
	Females	504.43 (502.41, 506.45)	196.49 (195.93, 197.05)	−3.2 (−3.3, −3.1)

Data are shown as estimates (95% confidence interval). Abbreviations: DALYs, disability-adjusted life years; YLDs, years lived with disability; AAPC, average annual percentage change.

**Table 2:** Average annual percentage change in age-standardized DALYs and YLDs rates attributable to nutritional deficiency among older adults (65+) from 1990 to 2019, by country in the Western Pacific region.

Burden	Economic Region	Country (in Descending Order of SDI in 2019)	Both Sexes	Male	Female
DALYs	High income	South Korea	−5.6 * (−5.8, −5.3)	−5.0 * (−5.5, −4.5)	−5.9 * (−6.0, −5.8)
		Japan	−1.2 * (−1.3, −1.2)	−0.6 * (−0.7, −0.4)	−1.4 * (−1.5, −1.4)
		Singapore	−3.2 * (−3.2, −3.2)	−3.0 * (−3.1, −2.9)	−3.3 * (−3.3, −3.3)
		New Zealand	−1.4 * (−1.7, −1.2)	−1.5 * (−1.8, −1.2)	−1.4 * (−1.8, −1.1)
		Australia	−1.0 * (−1.1, −0.9)	−1.3 * (−1.4, −1.1)	−0.8 (−0.9, −0.8)
		Brunei	−2.4 * (−2.8, −2.1)	−2.1 * (−2.4, −1.7)	−2.5 * (−2.7, −2.2)
		Cook Islands	−1.1 * (−1.2, −0.9)	−1.6 * (−1.7, −1.5)	−1.1 * (−1.2, −1.0)
		Palau	−0.9 * (−1.0, −0.9)	−1.3 * (−1.5, −1.2)	−0.9 * (−1.0, −0.9)
	Upper-middle income	Malaysia	−1.9 * (−2.2, −1.6)	−1.7 * (−2.0, −1.4)	−2.0 * (−2.3, −1.7)
		Niue	−1.6 * (−1.7, −1.5)	−2.1 * (−2.3, −1.9)	−1.4 (−1.5, −1.3)
		China	−3.1 * (−4.3, −1.9)	−2.3 * (−2.5, −2.1)	−3.6 * (−3.9, −3.4)
		Fiji	−0.9 * (−1.0, −0.8)	−0.8 * (−1.0, −0.7)	−0.9 * (−1.2, −0.7)
		Samoa	−0.9 * (−1.0, −0.8)	−1.5 * (−1.7, −1.4)	−0.6 * (−0.7, −0.5)
		Tonga	−0.8 * (−0.9, −0.7)	−1.2 * (−1.6, −0.1)	−0.8 * (−1.0, −0.6)
		Tuvalu	−1.8 * (−1.9, −1.8)	−2.7 * (−2.8, −2.6)	−1.3 * (−1.4, −1.2)
		Marshall Islands	−1.9 * (−2.1, −1.7)	−2.7 * (−3.0, −2.4)	−1.1 * (−1.2, −1.1)
	Lower-middle income	Philippines	−2.0 * (−2.3, −1.7)	−2.1 * (−2.5, −1.8)	−2.0 * (−2.4, −1.7)
		Nauru	−0.9 * (−1.1, −0.8)	−1.4 * (−1.5, −1.3)	−0.9 * (−1.0, −0.8)
		Vietnam	−3.4 * (−3.5, −3.3)	−4.0 * (−4.2, −3.9)	−2.7 * (−2.8, −2.7)
		Mongolia	−1.7 * (−1.8, −1.6)	−1.8 * (−2.0, −1.7)	−1.6 * (−1.7, −1.5)
		Federated States of Micronesia	−1.3 * (−1.4, −1.3)	−2.2 * (−2.3, −1.1)	−1.2 * (−1.2, −1.1)
		Kiribati	−1.1 * (−1.1, −1.0)	−1.7 * (−1.8, −1.6)	−1.0 * (−1.0, −0.9)
		Laos	−3.6 * (−3.7, −3.5)	−4.3 * (−4.5, −4.1)	−3.1 * (−3.2, −3.0)
		Vanuatu	−0.9 * (−1.0, −0.8)	−1.5 * (−1.6, −1.4)	−0.7 * (−0.8, −0.6)
		Cambodia	−5.0 * (−5.1, −4.9)	−5.9 * (−5.9, −5.8)	−4.2 * (−4.3, −4.1)
		Solomon Islands	−1.1 (−1.3, −1.0)	−2.1 * (−2.2, −2.0)	−0.8 * (−0.9, −0.8)
		Papua New Guinea	−1.1 * (−1.3, −0.9)	−1.9 * (−2.3, −1.6)	−0.6 * (−0.7, −0.5)
YLDs	High income	South Korea	−5.1 * (−5.3, −5.0)	−4.4 * (−4.6, −4.2)	−5.5 * (−5.7, −5.4)
		Japan	−3.0 * (−3.0, −3.0	−2.7 * (−2.8, −2.7)	−3.1 * (−3.1, −3.1)
		Singapore	−3.0 * (−3.0, −3.0)	−2.7 * (−2.8, −2.7)	−3.1 * (−3.1, −3.1)
		New Zealand	−1.2 * (−1.3, −1.0)	−0.9 * (−1.2, −0.6)	−1.2 * (−1.3, −1.0)
		Australia	−0.9 * (−1.0, −0.8)	−1.0 * (−1.3, −0.6)	−0.8 * (−0.9, −0.7)
		Brunei	−1.6 * (−1.6, −1.6)	−1.6 * (−1.7, −1.6)	−1.6 * (−1.7, −1.5)
		Cook Islands	−0.9 * (−1.0, −0.8)	−1.4 * (−1.5, −1.3)	−0.9 * (−1.0, −0.8
		Palau	−0.9 * (−1.0, −0.8	−1.3 * (−1.5, −1.1)	−0.8 * (−0.9, −0.7)
	Upper-middle income	Malaysia	−2.1 * (−2.2, −1.9)	−1.7 * (−1.9, −1.5)	−2.4 * (−2.5, −2.3)
		Niue	−1.1 * (−1.3, −1.0)	−1.5 * (−1.9, −1.1)	−1.0 * (−1.2, −0.8)
		China	−3.2 * (−3.3, −3.1)	−2.6 * (−2.8, −2.4)	−3.7 * (−3.8, −3.6)
		Fiji	0.1 * (0.0, 0.2)	0.1 (−0.1, 0.2)	−0.1 * (−0.2, −0.1)
		Samoa	−0.5 * (−0.7, −0.4)	−0.8 * (−1.0, −0.6)	−0.4 * (−0.6, −0.3)
		Tonga	−0.4 * (−0.4, −0.3)	−1.0 * (−1.3, −0.7)	−0.4 * (−0.5, −0.3)
		Tuvalu	−1.0 * (−1.1, −1.0)	−1.5 * (−1.6, −1.4)	−0.7 * (−0.8, −0.7)
		Marshall Islands	−0.8 * (−0.9, −0.7)	−0.9 * (−1.0, −0.8)	−0.4 * (−0.4, −0.3)
	Lower-middle income	Philippines	−1.8 * (−1.8, −1.7)	−2.0 * (−2.1, −1.9)	−1.7 * (−1.8, −1.7)
		Nauru	−0.4 * (−0.5, −0.2)	−1.1 * (−1.3, −0.9)	−0.5 * (−0.5, −0.4)
		Vietnam	−2.6 * (−2.7, −2.5)	−2.5 * (−2.6, −2.3)	−2.6 * (−2.6, −2.6)
		Mongolia	−1.6 * (−1.7, −1.5)	−1.7 * (−1.7, −1.6)	−1.6 * (−1.7, −1.5)
		Federated States of Micronesia	−0.7 * (−1.0, −0.4)	−1.5 * (−1.7, −1.3)	−0.7 * (−0.7, −0.6)
		Kiribati	−0.3 * (−0.3, −0.2)	−0.9 * (−1.0, −0.8)	−0.2 * (−0.3, −0.2)
		Laos	−1.8 * (−1.9, −1.8)	−1.9 * (−1.9, −1.8)	−1.8 * (−1.9, −1.7)
		Vanuatu	0.1 * (0.0, 0.2)	−0.2 * (−0.4, −0.0)	0.0 (−0.0, 0.1)
		Cambodia	−2.0 * (−2.1, −1.9)	−2.1 * (−2.3, −1.9)	−2.0 * (−2.1, −1.9)
		Solomon Islands	−0.2 * (−0.3, −0.1)	−0.9 * (−1.0, −0.8)	−0.4 * (−0.4, −0.3)
		Papua New Guinea	−0.3 * (−0.4, −0.2)	−0.7 * (−0.8, −0.6)	−0.1 * (−0.2, −0.1)

Data are shown as estimates (95% confidence interval); * indicates that the *p*-value < 0.05. Abbreviations: DALYs, disability-adjusted life years; YLDs, years lived with disability; AAPC, average annual percentage change.

**Table 3:** Associations of age-standardized DALYs and YLDs attributable to nutritional deficiency with sex and SDI among older adults (65+) from 1990 to 2019 in the Western Pacific region.

Burden	Sex	Mean (95% CI)	Spearman Correlation with SDI (95% CI)
DALYs	Both sexes	1038.25 (981.45, 1095.05)	−0.899 ** (−0.911, −0.883)
	Male	767.31 (721.45, 823.29)	−0.904 ** (−0.913, −0.893)
	Female	1273.75 (120.8.08, 1345.67)	−0.847 ** (−0.867, −0.824)
YLDs	Both sexes	503.27 (487.02, 519.52)	−0.855 ** (−0.875, −0.830)
	Male	298.16 (287.41, 310.15)	−0.682 ** (−0.729, −0.631)
	Female	685.92 (661.83, 709.61)	−0.844 ** (−0.864, −0.820)

Pooled analyses using data from all years/countries; ** indicates *p*-value < 0.01. Abbreviations: DALYs, disability-adjusted life years; YLDs, years lived with disability; SDI, social development index.

**Table 4:** Results of the mixed-effects regression on age-standardized DALYs and YLDs rates attributed to nutritional deficiency on sex, SDI, and sex by SDI, among older adults from 1990 to 2019 in the Western Pacific region.

Factor	DALYs	YLDs
Sex (Reference = female)	−1273.13 (−1417.43, −1128.83)	−1095.58 (−1144.96, −1046.19)
SDI	−6228.80 (−7144.95, −5312.64)	−2303.50 (−2551.79, −2055.20)
Sex × SDI	1317.44 (1077.74, 1557.14)	1216.17 (1134.13, 1298.20)

Models have controlled for time and country. Data are shown as coefficients (95% confidence interval). Abbreviations: DALYs, disability-adjusted life years; YLDs, years lived with disability; SDI, social development index.

## Data Availability

Data are available in a public, open access repository. All of the data are publically available. Data are available on request.

## Supplementary Materials

The following are available online at https://www.mdpi.com/article/10.3390/nu13124421/s1, Figure S1: Average annual percentage change (AAPC) in the age-standardized rates of the DALYs and YLDs attributed to nutritional deficiency among older adults (≥65 years) between 1990 and 2019 in both sexes and by country in the Western Pacific region, Table S1: Age-specific numbers and crude rates in DALYs and YLDs among older adults, stratified by sex, in the Western Pacific region.
