# Supplementary material for: Magnitude, Temporal Trends, and Inequalities in the DALYs and YLDs of Nutritional Deficiency among Older Adults in the Western Pacific Region: Findings from the Global Burden of Disease Study 1990–2019
Source: Nutrients. 2021 Dec 10;13(12):4421. doi: 10.3390/nu13124421 (PMC8706447; doi:10.3390/nu13124421)
Supplement: Supplementary file 1 [file nutrients-13-04421-s001.zip › Supplementary Figure S1 (7Dec2021).pdf]

Supplementary Figure S1. Average annual percentage change (AAPC) of in the age-standardized rates of the DALYs and YLDs attributed to nutritional deficiency among older adults (≥ 65 years) between 1990 and 2019 in both sexes and by country in the Western Pacific region.

<table>
<tr><th colspan="4">A. Age-standardized rates in DALYs</th></tr>
<tr><td>Region / Country (SDI in 2019)</td><td>Both Sex</td><td>Male</td><td>Female</td></tr>
<tr><td colspan="4">High income economies ($12536 or more)</td></tr>
<tr><td>1. South Korea (0.878)</td>
<td>AAPC = -5.6* (-5.8, -5.3)
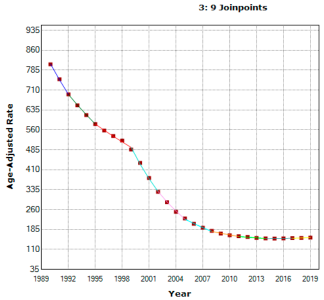</td>
<td>AAPC = -5.0* (-5.5, -4.5)
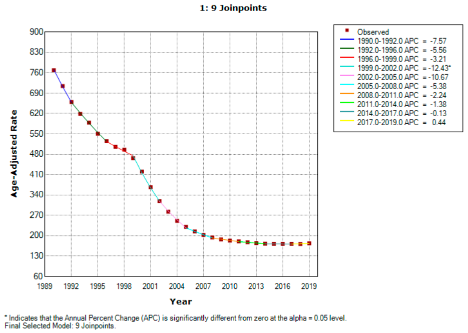</td>
<td>AAPC = -5.9* (-6.0, -5.8)
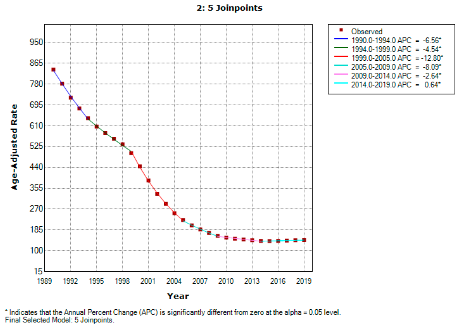</td></tr>
<tr><td>2. Japan (0.87)</td>
<td>AAPC = -1.2* (-1.3, -1.2)
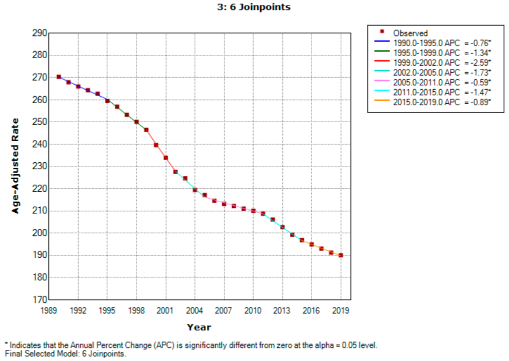</td>
<td>AAPC = -0.6* (-0.7, -0.4)
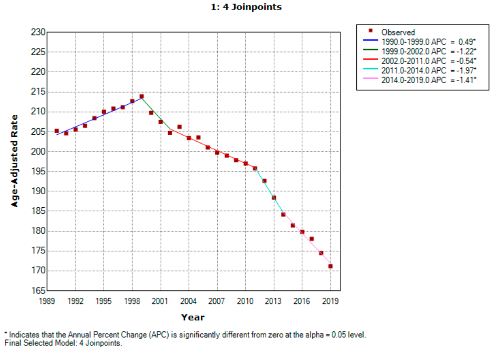</td>
<td>AAPC = -1.4* (-1.5, -1.4)
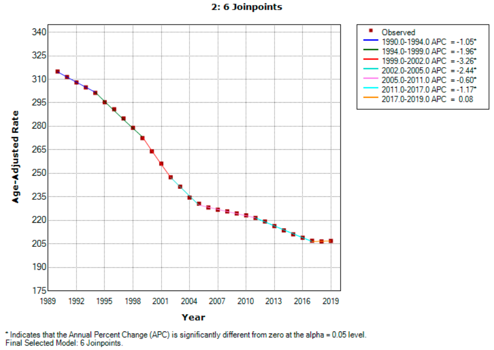</td></tr>
</table>

<table>
<tr>
<td>3. Singapore (0.861)</td>
<td>AAPC = -3.2* (-3.2, -3.2)
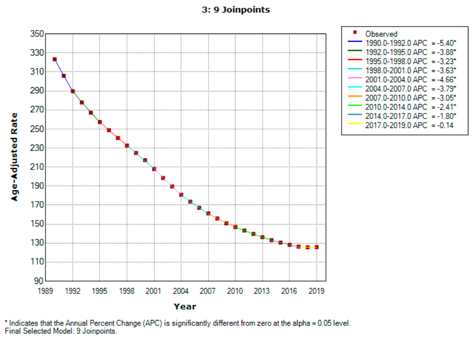
</td>
<td>AAPC = -3.0* (-3.1, -2.9)
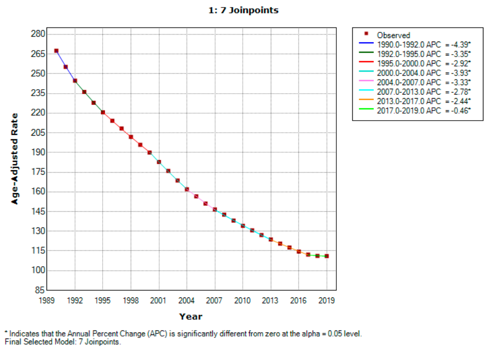
</td>
<td>AAPC = -3.3* (-3.3, -3.3)
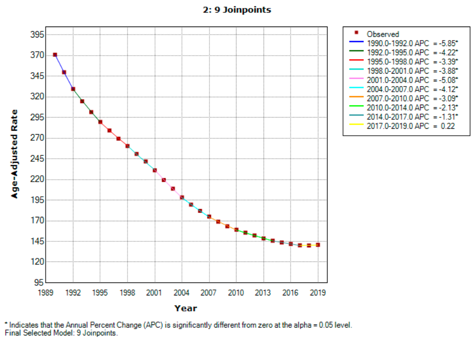
</td>
</tr>
<tr>
<td>4. New Zealand (0.84)</td>
<td>AAPC = -1.4* (-1.7, -1.2)
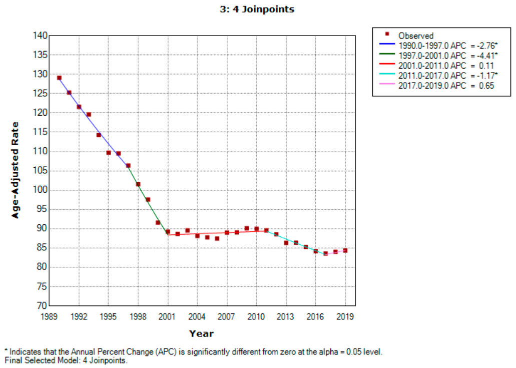
</td>
<td>AAPC = -1.5* (-1.8, -1.2)
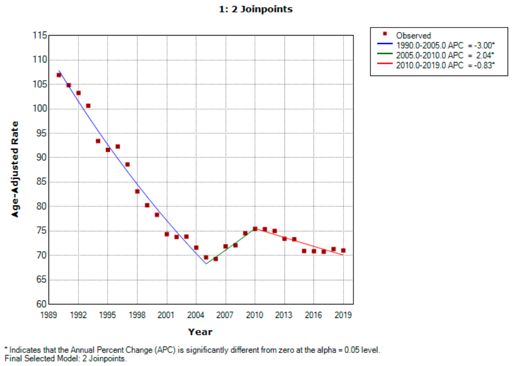
</td>
<td>AAPC = -1.4* (-1.8, -1.1)
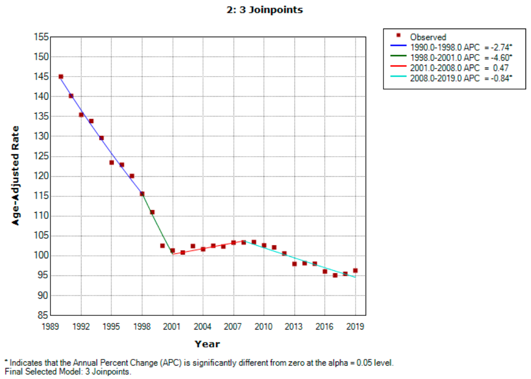
</td>
</tr>
<tr>
<td>5. Australia (0.839)</td>
<td>AAPC = -1.0* (-1.1, -0.9)
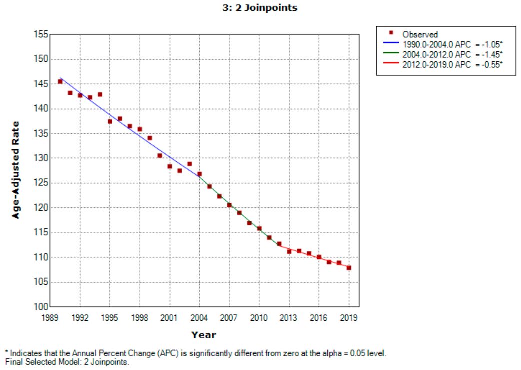
</td>
<td>AAPC = -1.3* (-1.4, -1.1)
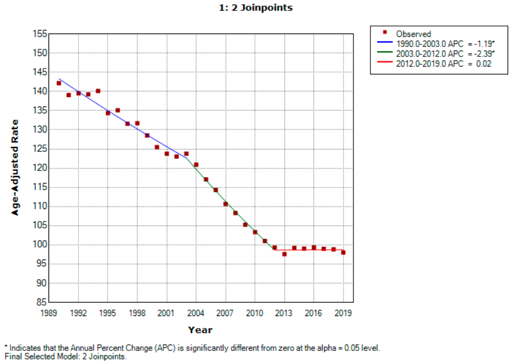
</td>
<td>AAPC = -0.8 (-0.9, -0.8)
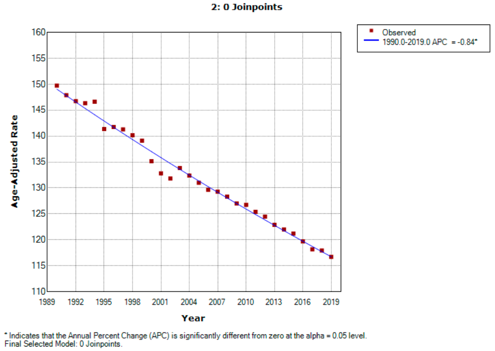
</td>
</tr>
</table>

<table>
<tr>
<td>6. Brunei (0.823)</td>
<td>AAPC = -2.4* (-2.8, -2.1)
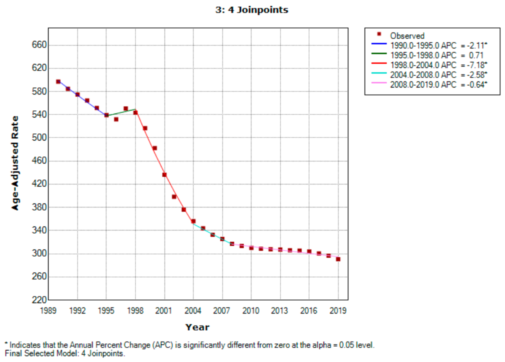
</td>
<td>AAPC = -2.1* (-2.4,-1.7)
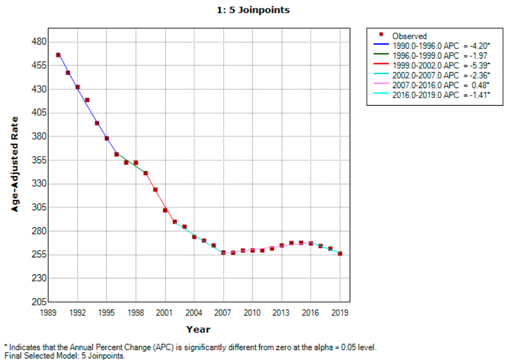
</td>
<td>AAPC = -2.5* (-2.7, -2.2)
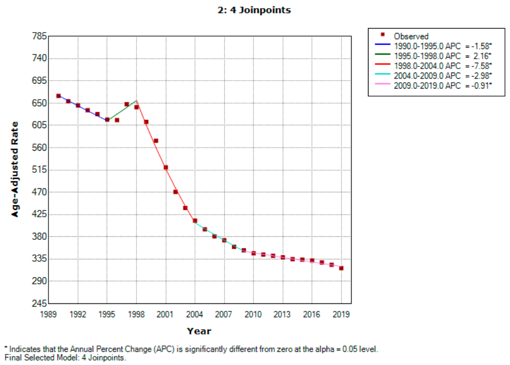
</td>
</tr>
<tr>
<td>7. Cook Islands (0.764)</td>
<td>AAPC = -1.1* (-1.2, -0.9)
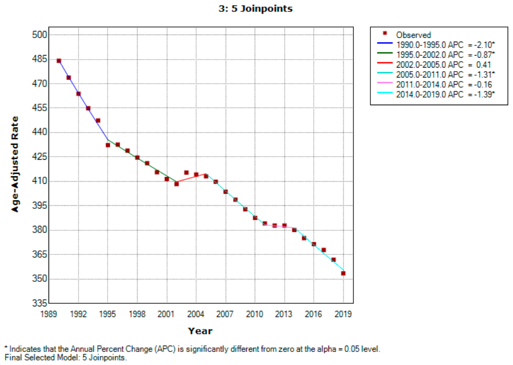
</td>
<td>AAPC = -1.6* (-1.7, -1.5)
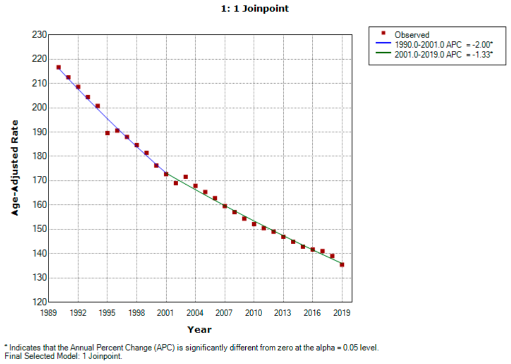
</td>
<td>AAPC = -1.1* (-1.2, -1.0)
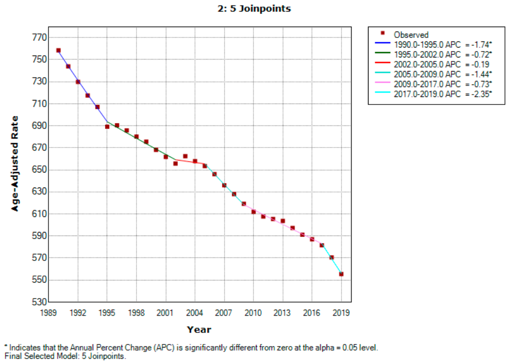
</td>
</tr>
<tr>
<td>8. Palau (0.738)</td>
<td>AAPC = -0.9* (-1.0, -0.9)
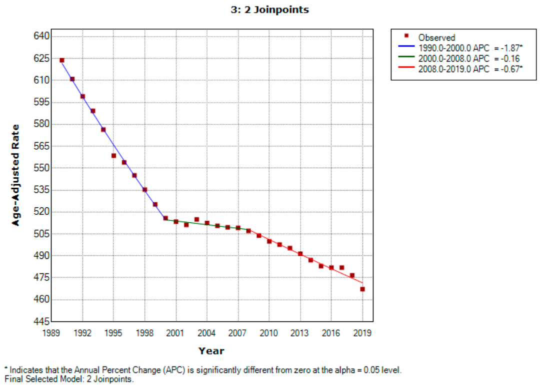
</td>
<td>AAPC = -1.3* (-1.5, -1.2)
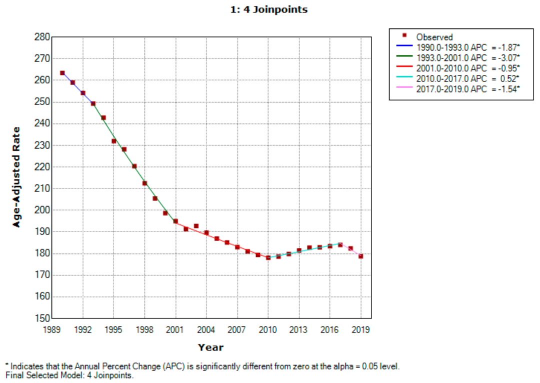
</td>
<td>AAPC = -0.9* (-1.0, -0.9)
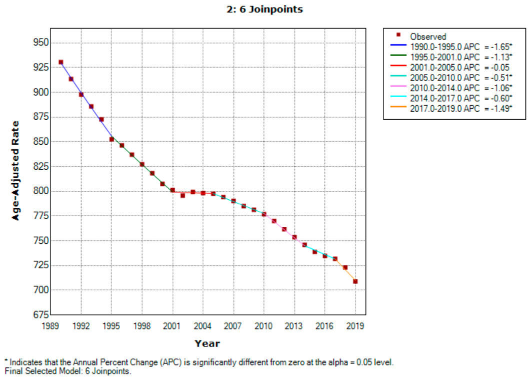
</td>
</tr>
</table>

Upper-Middle income economies ($4046 to $12535)

9. Malaysia (0.737)

AAPC = -1.9* (-2.2, -1.6)

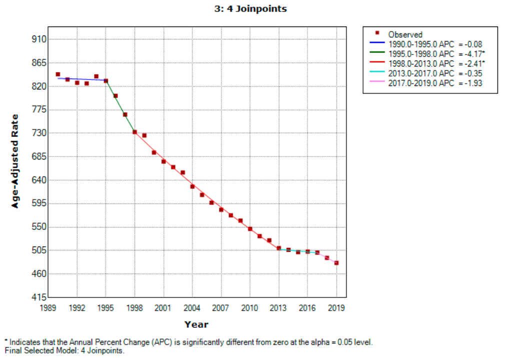

AAPC = -1.7* (-2.0, -1.4)

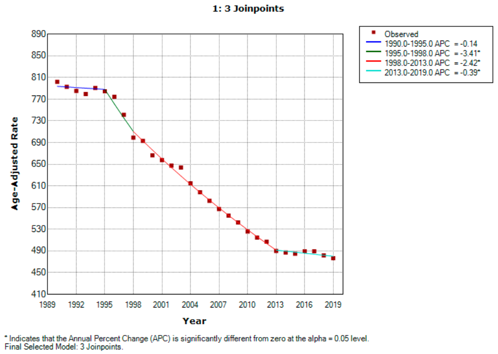

AAPC = -2.0* (-2.3, -1.7)

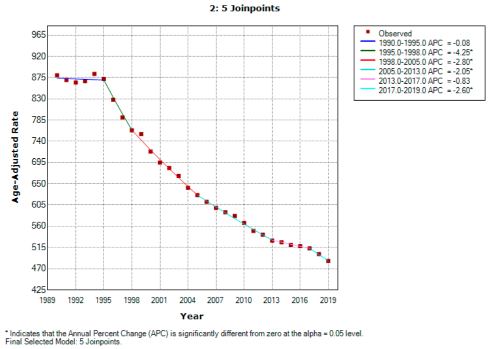

10. Niue (0.711)

AAPC = -1.6* (-1.7, -1.5)

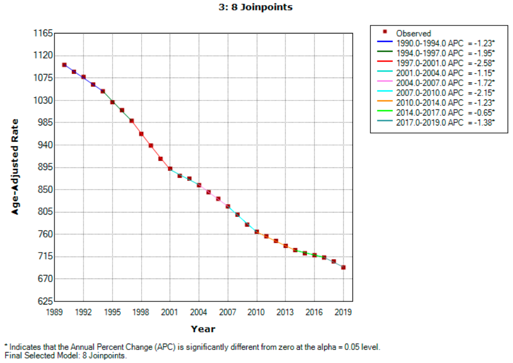

AAPC = -2.1* (-2.3, -1.9)

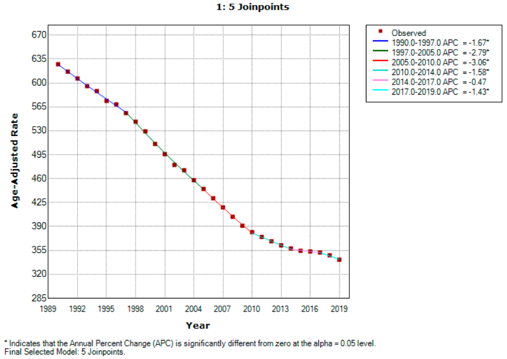

AAPC = -1.4 (-1.5, -1.3)

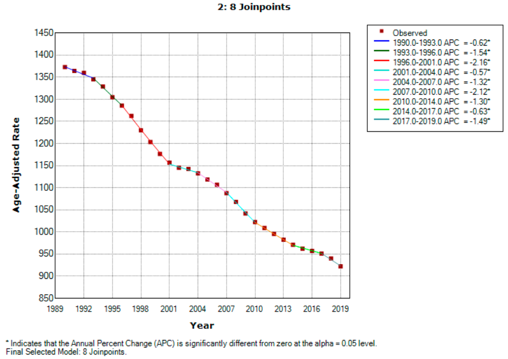

11. China (0.686)

AAPC = -3.1* (-4.3, -1.9)

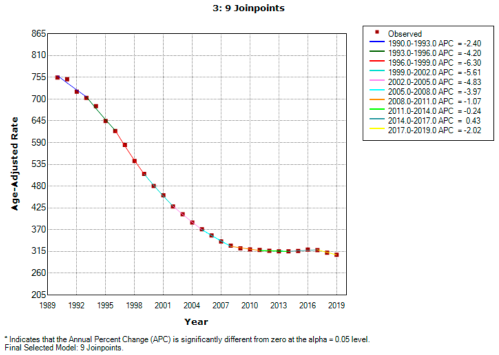

AAPC = -2.3* (-2.5, -2.1)

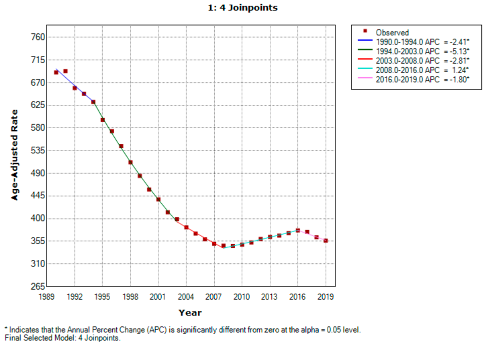

AAPC = -3.6* (-3.9, -3.4)

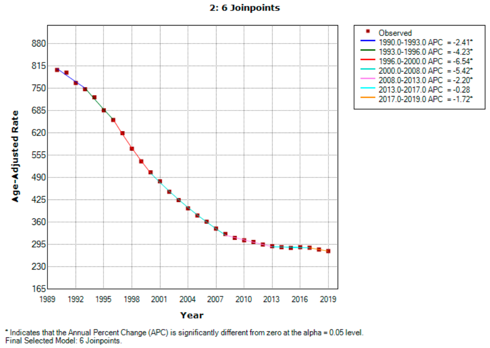

<table>
<tr>
<td>12. Fiji (0.664)</td>
<td>AAPC = -0.9* (-1.0, -0.8)
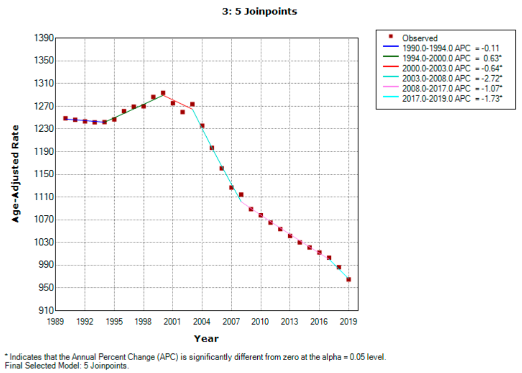
</td>
<td>AAPC = -0.8* (-1.0, -0.7)
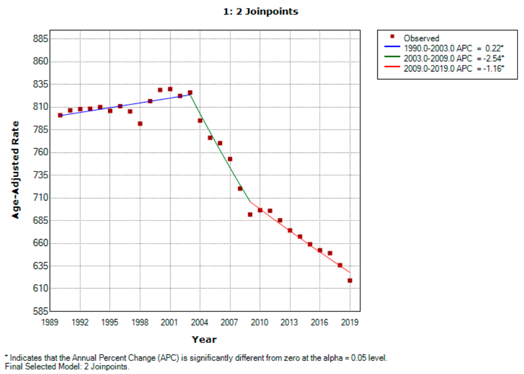
</td>
<td>AAPC = -0.9* (-1.2, -0.7)
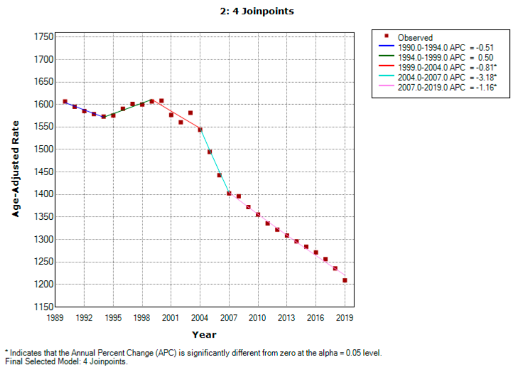
</td>
</tr>
<tr>
<td>13. Samoa (0.641)</td>
<td>AAPC = -0.9* (-1.0, -0.8)
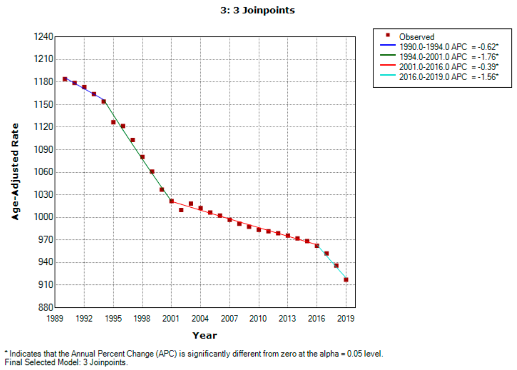
</td>
<td>AAPC = -1.5* (-1.7, -1.4)
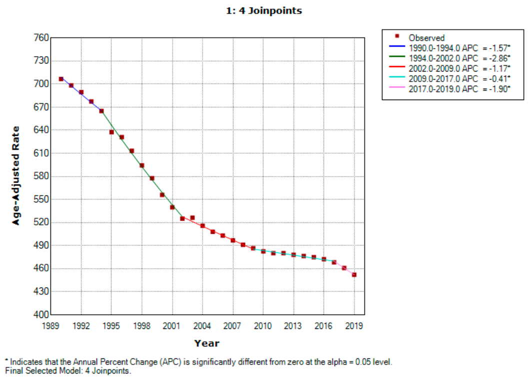
</td>
<td>AAPC = -0.6* (-0.7, -0.5)
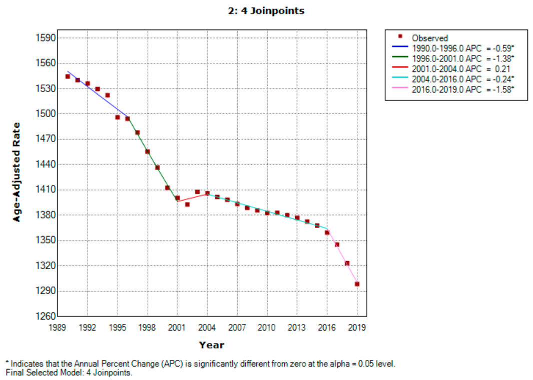
</td>
</tr>
<tr>
<td>14. Tonga (0.636)</td>
<td>AAPC = -0.8* (-0.9, -0.7)
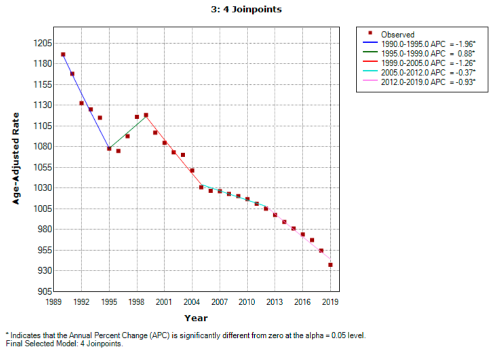
</td>
<td>AAPC = -1.2* (-1.6, -0.1)
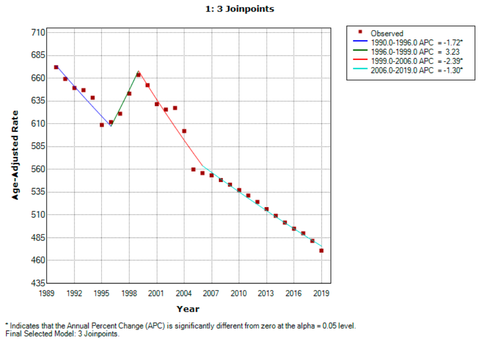
</td>
<td>AAPC = -0.8* (-1.0, -0.6)
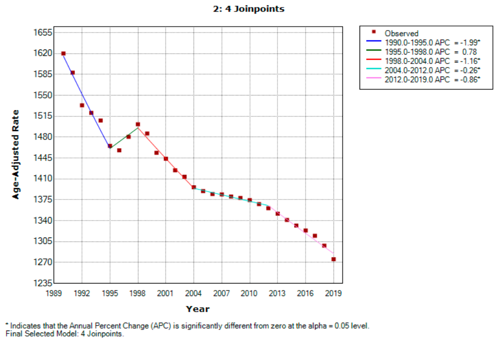
</td>
</tr>
</table>

<table>
<tr>
<td>19. Tuvalu (0.589)</td>
<td>AAPC = -1.8* (-1.9, -1.8)
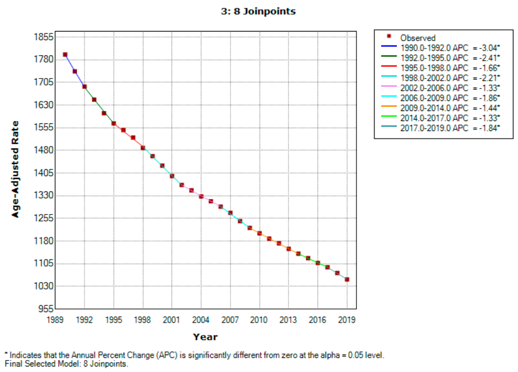
</td>
<td>AAPC = -2.7* (-2.8, -2.6)
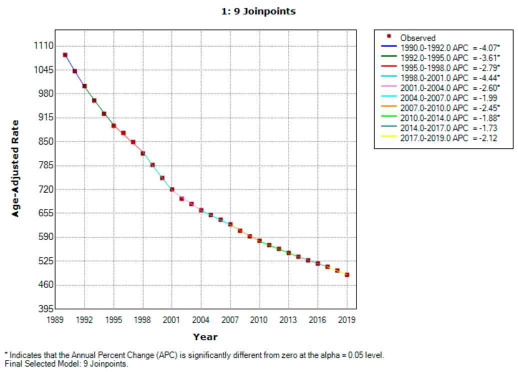
</td>
<td>AAPC = -1.3* (-1.4, -1.2)
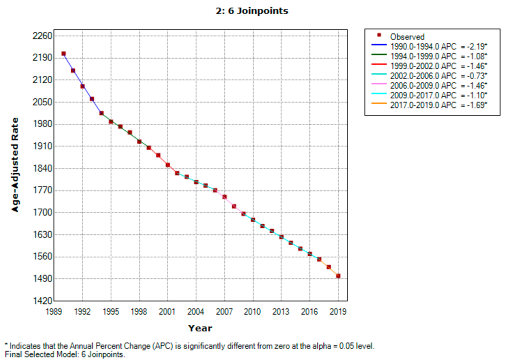
</td>
</tr>
<tr>
<td>21. Marshall Islands (0.544)</td>
<td>AAPC = -1.9* (-2.1, -1.7)
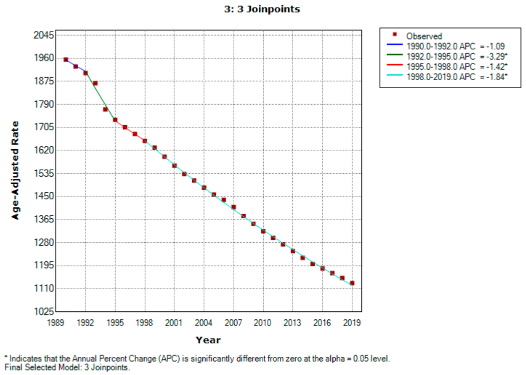
</td>
<td>AAPC = -2.7* (-3.0, -2.4)
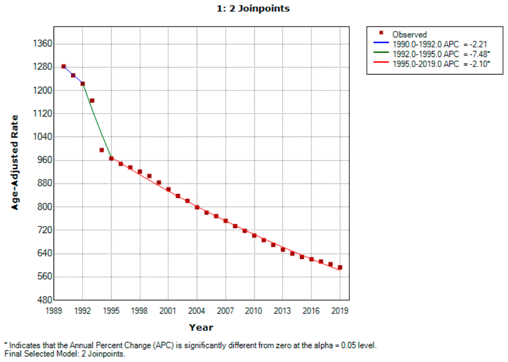
</td>
<td>AAPC = -1.1* (-1.2, -1.1)
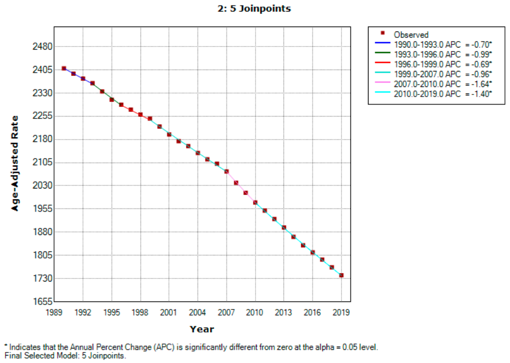
</td>
</tr>
<tr>
<td></td><td></td><td></td><td></td>
</tr>
</table>

Lower-Middle income economies ($1036 to $4045)

15. Philippines (0.623)

AAPC = -2.0* (-2.3, -1.7)

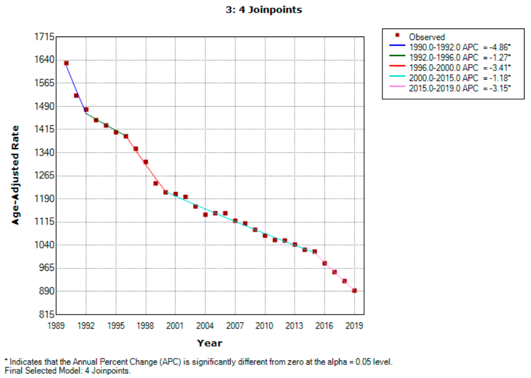

AAPC = -2.1* (-2.5, -1.8)

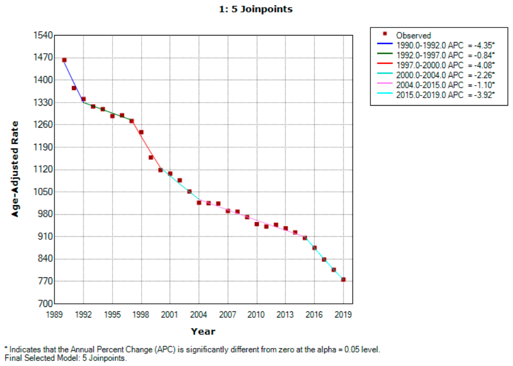

AAPC = -2.0* (-2.4, -1.7)

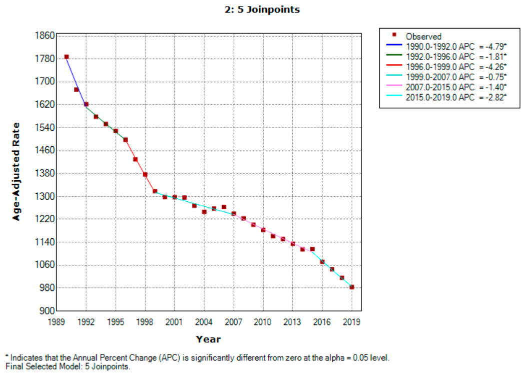

16. Nauru (0.618)

AAPC = -0.9* (-1.1, -0.8)

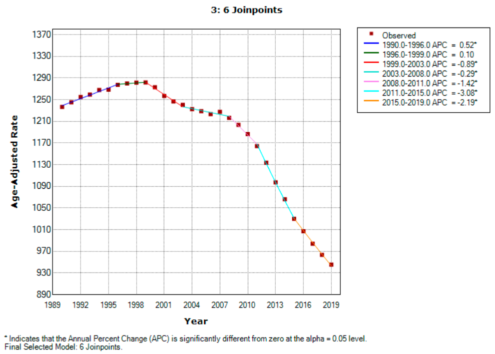

AAPC = -1.4* (-1.5, -1.3)

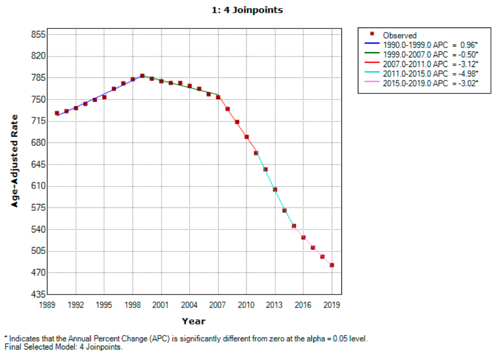

AAPC = -0.9* (-1.0, -0.8)

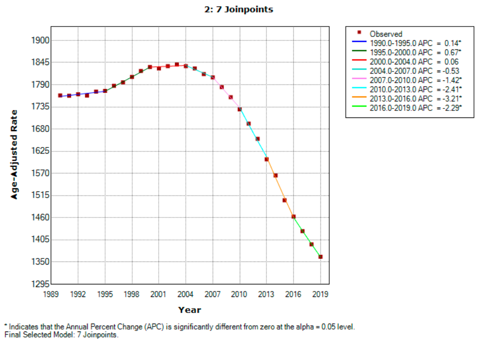

17. Vietnam (0.617)

AAPC = -3.4* (-3.5, -3.3)

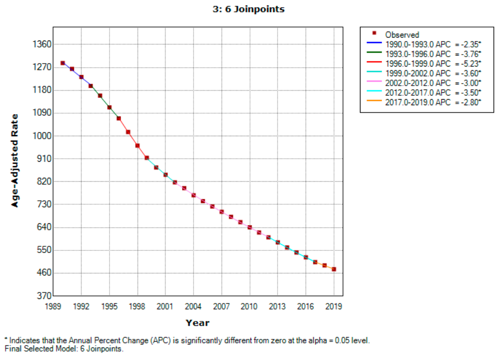

AAPC = -4.0* (-4.2, -3.9)

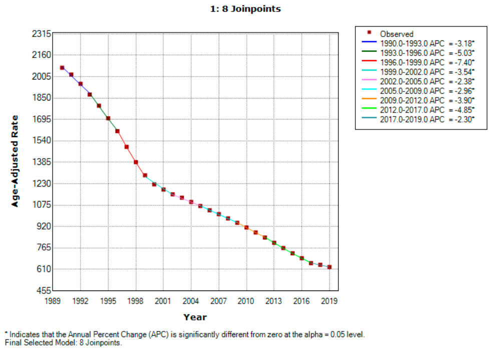

AAPC = -2.7* (-2.8, -2.7)

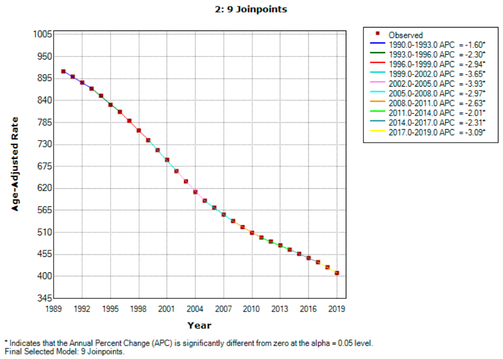

18. Mongolia (0.606)

AAPC = -1.7* (-1.8, -1.6)

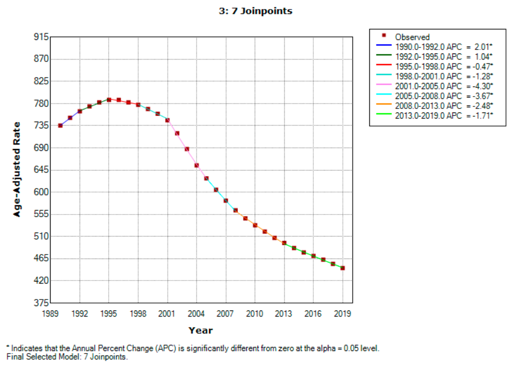

AAPC = -1.8* (-2.0, -1.7)

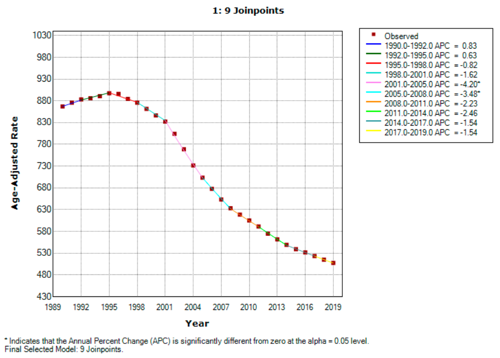

AAPC = -1.6* (-1.7, -1.5)

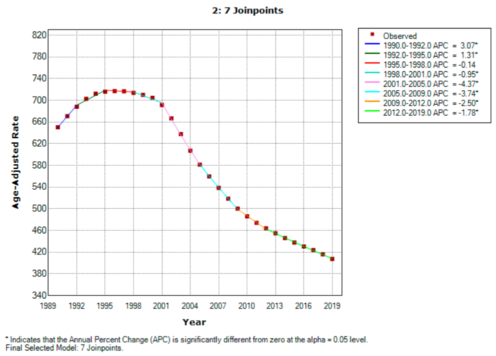

20. Federated States of Micronesia (0.580)

AAPC = -1.3* (-1.4, -1.3)

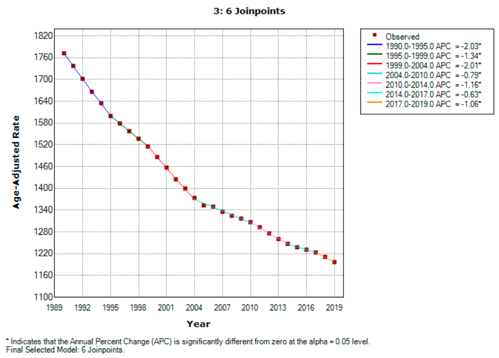

AAPC = -2.2* (-2.3, -1.1)

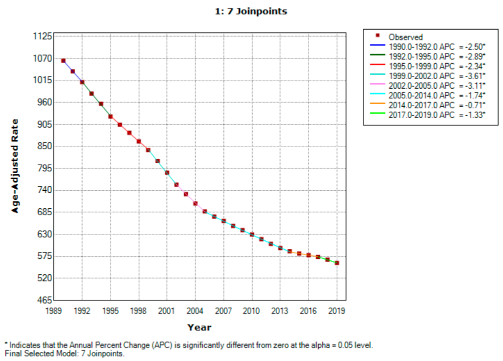

AAPC = -1.2* (-1.2, -1.1)

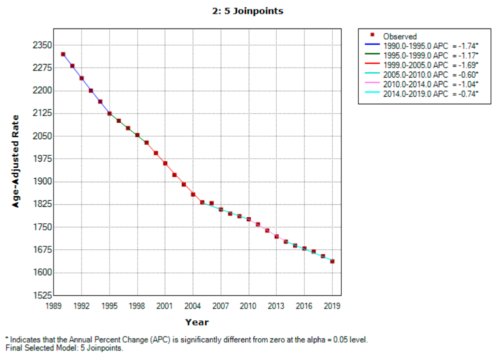

22. Kiribati (0.527)

AAPC = -1.1* (-1.1, -1.0)

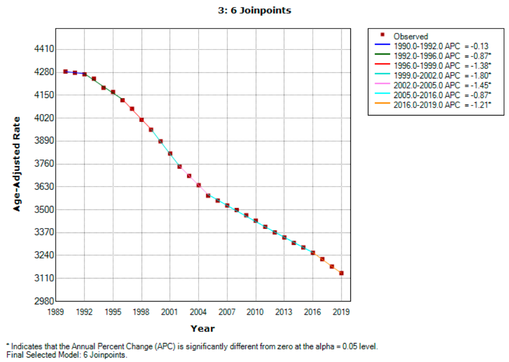

AAPC = -1.7* (-1.8, -1.6)

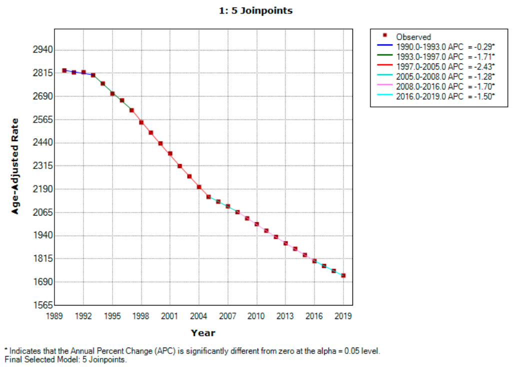

AAPC = -1.0* (-1.0, -0.9)

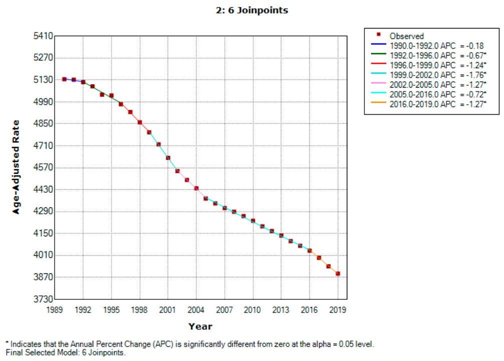

23. Laos (0.49)	AAPC = -3.6* (-3.7, -3.5)<br>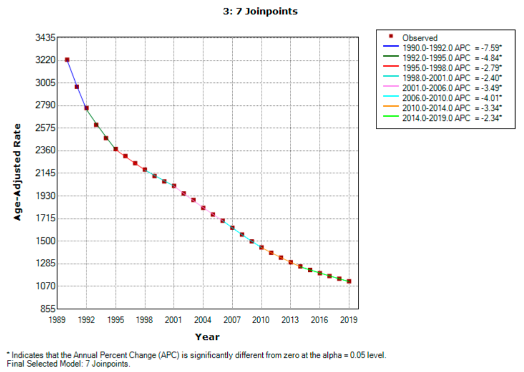 	AAPC = -4.3* (-4.5, -4.1)<br>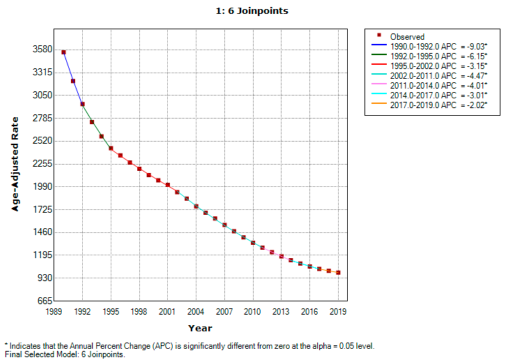 	AAPC = -3.1* (-3.2, -3.0)<br>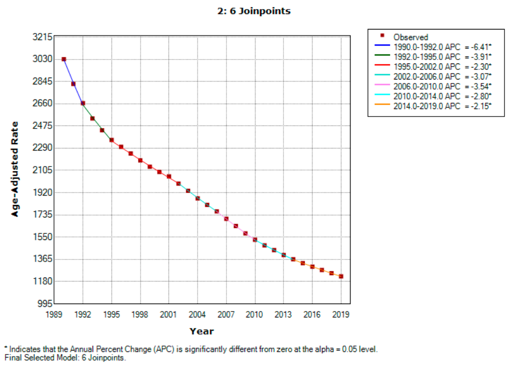
24. Vanuatu (0.485)	AAPC = -0.9* (-1.0, -0.8)<br>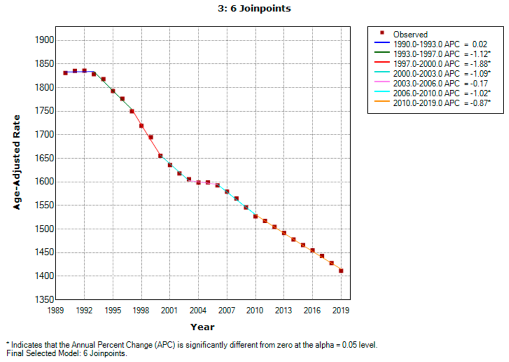 	AAPC = -1.5* (-1.6, -1.4)<br>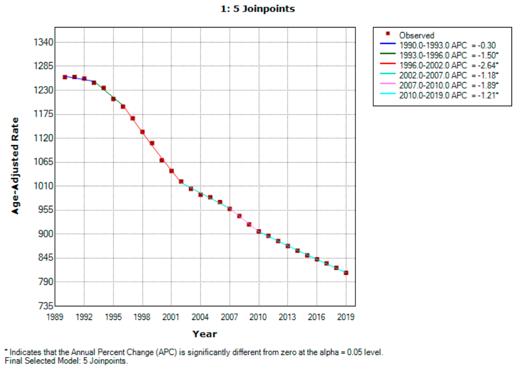 	AAPC = -0.7* (-0.8, -0.6)<br>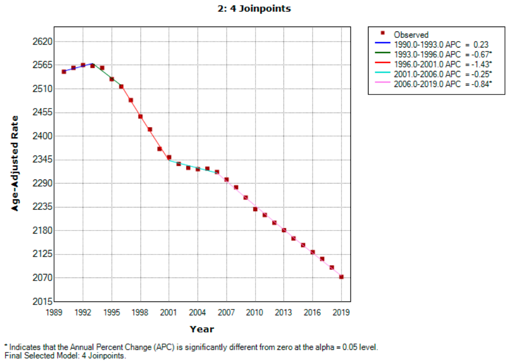
25. Cambodia (0.469)	AAPC = -5.0* (-5.1, -4.9)<br>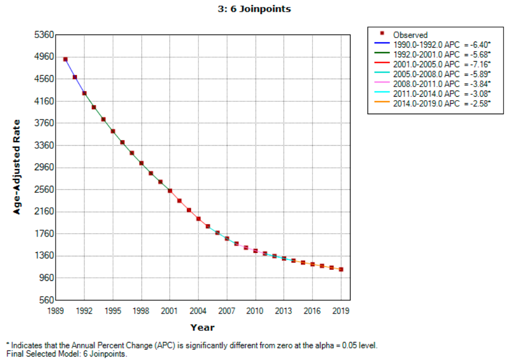 	AAPC = -5.9* (-5.9, -5.8)<br>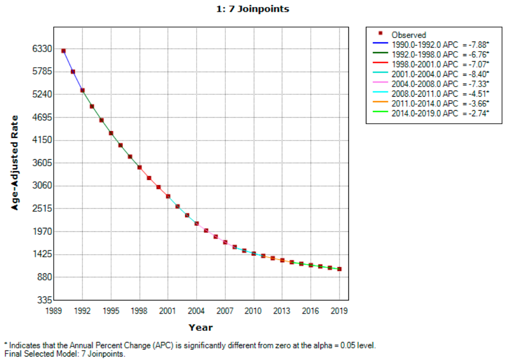 	AAPC = -4.2* (-4.3, -4.1)<br>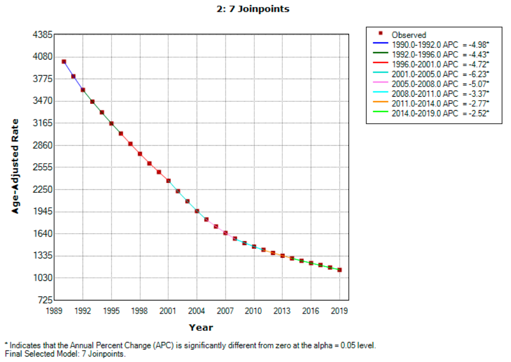

26. Solomon Islands (0.407)

AAPC = -1.1 (-1.3, -1.0)

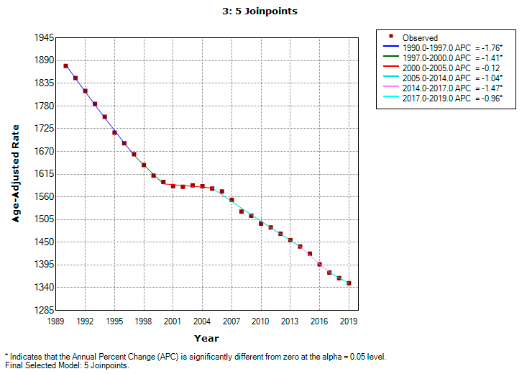

AAPC = -2.1* (-2.2, -2.0)

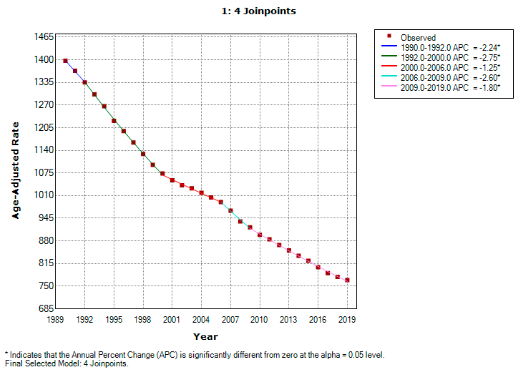

AAPC = -0.8* (-0.9, -0.8)

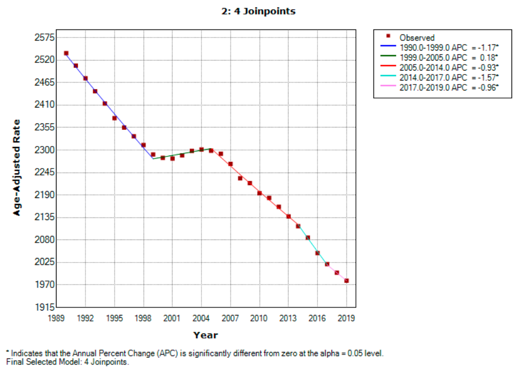

27. Papua New Guinea (0.394)

AAPC = -1.1* (-1.3, -0.9)

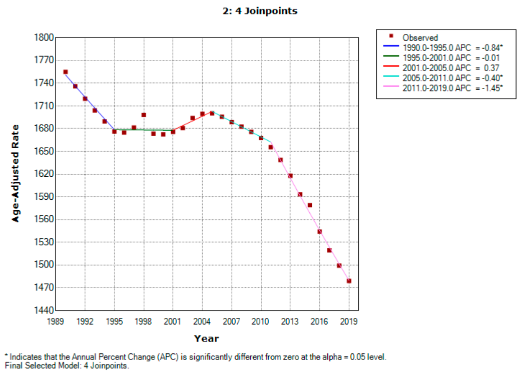

AAPC = -1.9* (-2.3, -1.6)

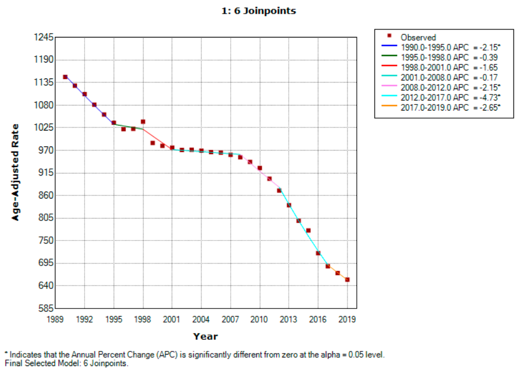

AAPC = -0.6* (-0.7, -0.5)

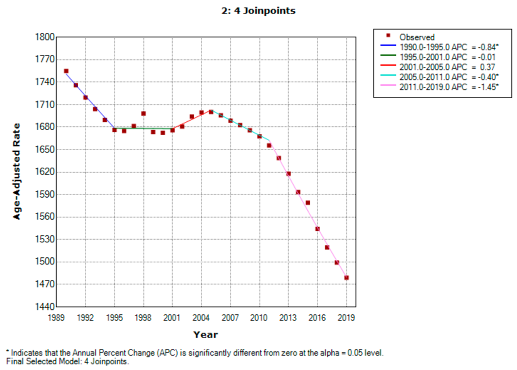

<table>
<tr><th colspan="4">B. Age-standardized rates in YLDs</th></tr>
<tr><th>Region / Country
(SDI in 2019)</th><th>Both Sex</th><th>Male</th><th>Female</th></tr>
<tr><td></td><td colspan="3">High income economies ($12536 or more)</td></tr>
<tr><td>1. South Korea (0.878)</td><td>AAPC = -5.1* (-5.3, -5.0)
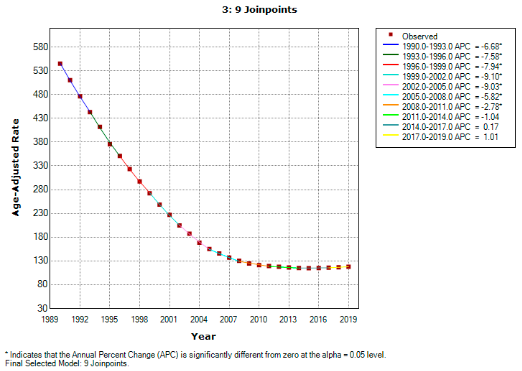
</td><td>AAPC = -4.4* (-4.6, -4.2)
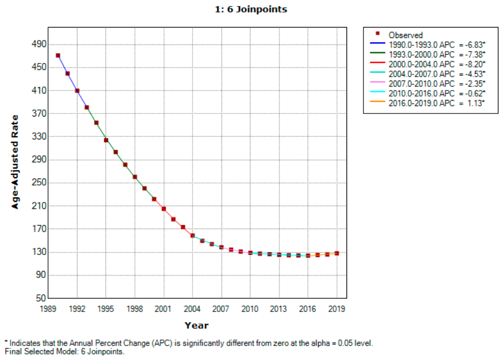
</td><td>AAPC = -5.5* (-5.7, -5.4)
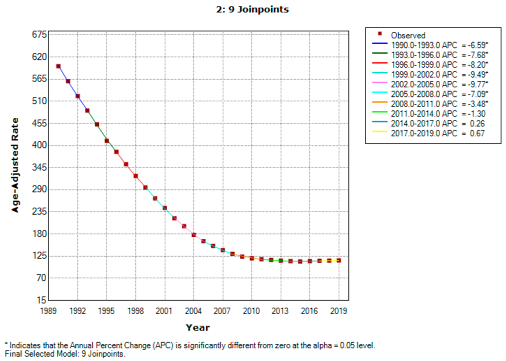
</td></tr>
<tr><td>2. Japan (0.87)</td><td>AAPC = -3.0* (-3.0, -3.0)
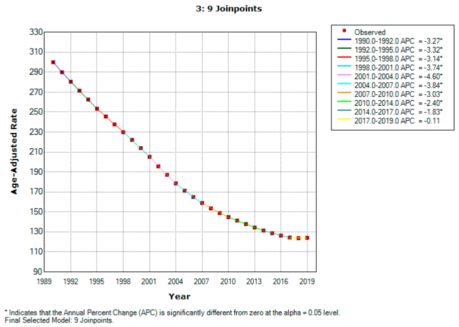
</td><td>AAPC = -2.7* (-2.8, -2.7)
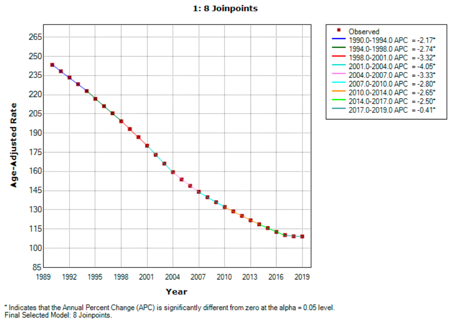
</td><td>AAPC = -3.1* (-3.1, -3.1)
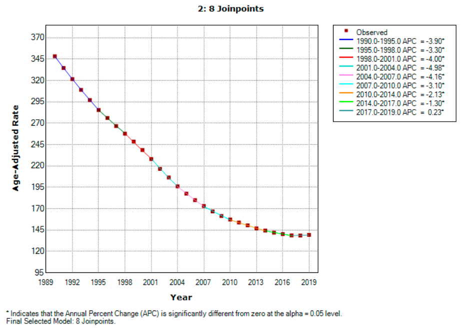
</td></tr>
</table>

3. Singapore (0.861)

AAPC = -3.0* (-3.0, -3.0)

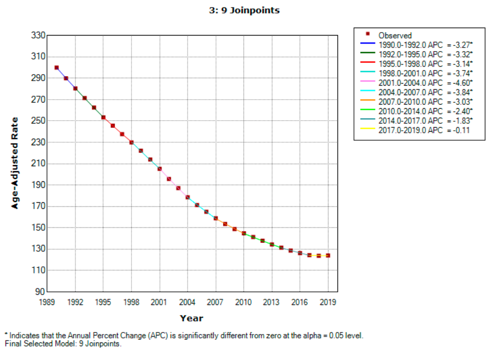

AAPC = -2.7* (-2.8, -2.7)

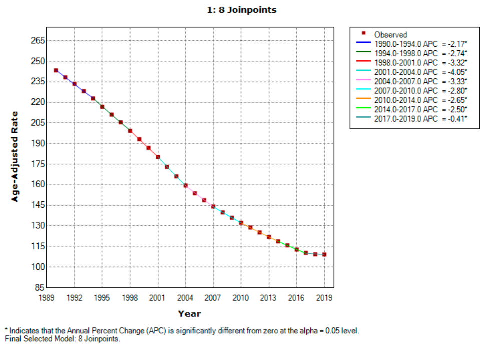

AAPC = -3.1* (-3.1, -3.1)

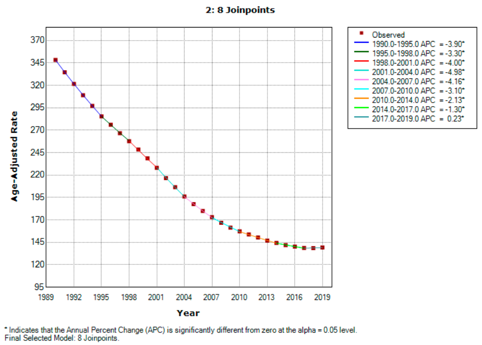

4. New Zealand (0.84)

AAPC = -1.2* (-1.3, -1.0)

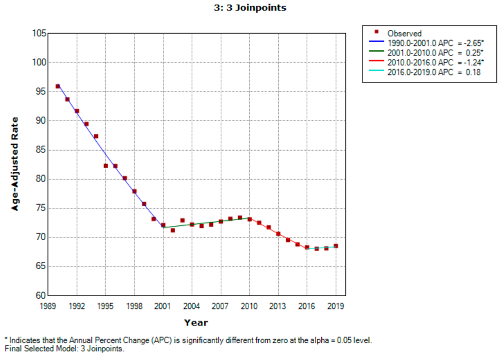

AAPC = -0.9* (-1.2, -0.6)

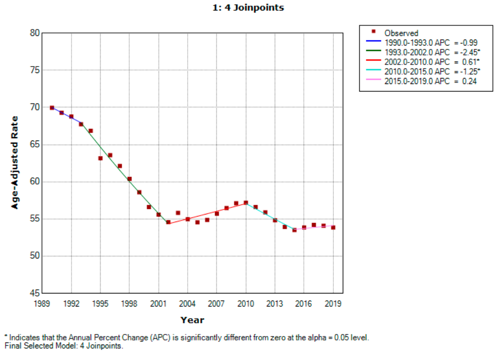

AAPC = -1.2* (-1.3, -1.0)

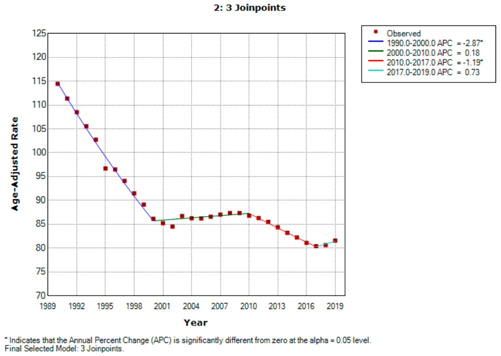

5. Australia (0.839)

AAPC = -0.9* (-1.0, -0.8)

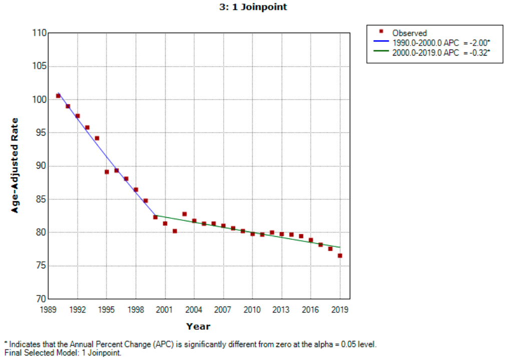

AAPC = -1.0* (-1.3, -0.6)

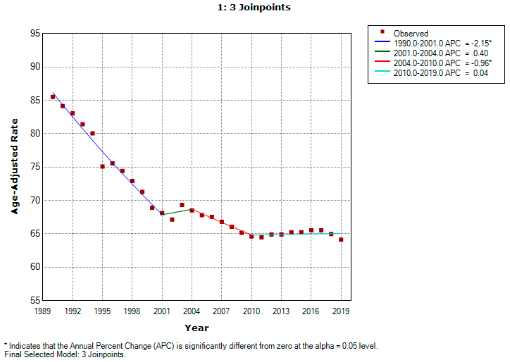

AAPC = -0.8* (-0.9, -0.7)

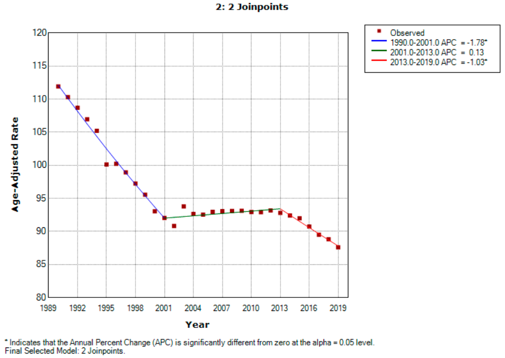

<table>
<tr>
<td>6. Brunei (0.823)</td>
<td>AAPC = -1.6* (-1.6, -1.6)
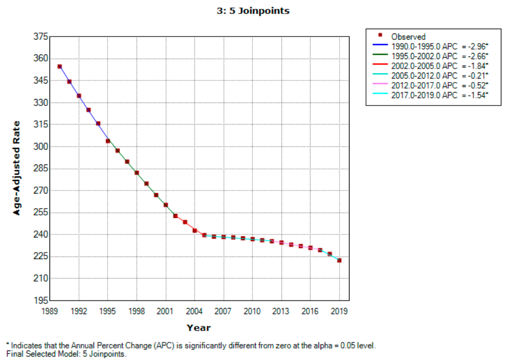
</td>
<td>AAPC = -1.6* (-1.7, -1.6)
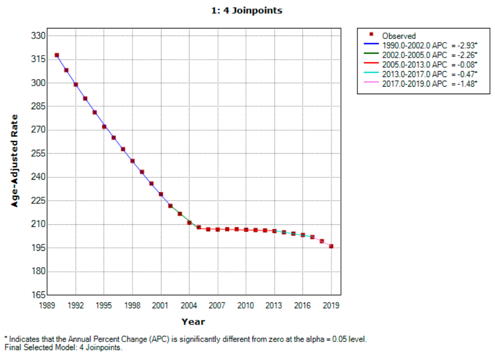
</td>
<td>AAPC = -1.6* (-1.7, -1.5)
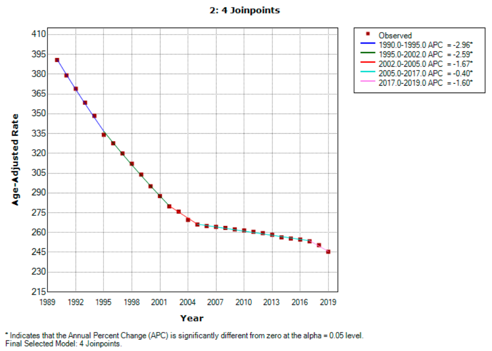
</td>
</tr>
<tr>
<td>7. Cook Islands (0.764)</td>
<td>AAPC = -0.9* (-1.0, -0.8)
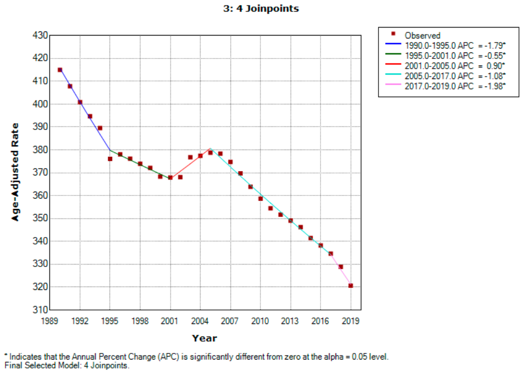
</td>
<td>AAPC = -1.4* (-1.5, -1.3)
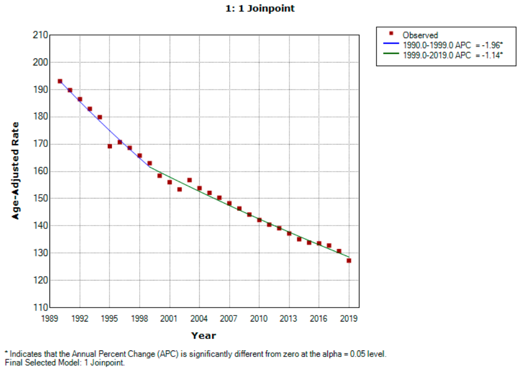
</td>
<td>AAPC = -0.9* (-1.0, -0.8)
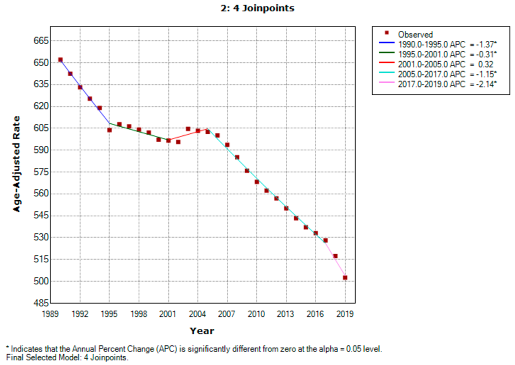
</td>
</tr>
<tr>
<td>8. Palau (0.738)</td>
<td>AAPC = -0.9* (-1.0, -0.8)
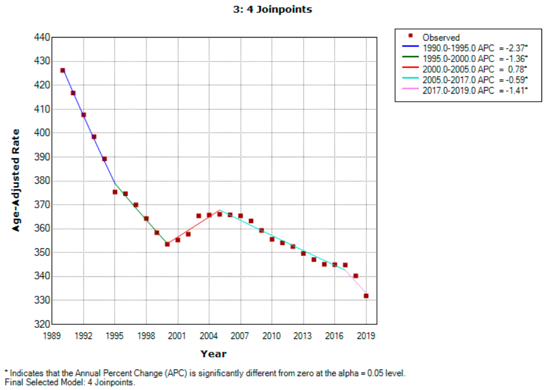
</td>
<td>AAPC = -1.3* (-1.5, -1.1)
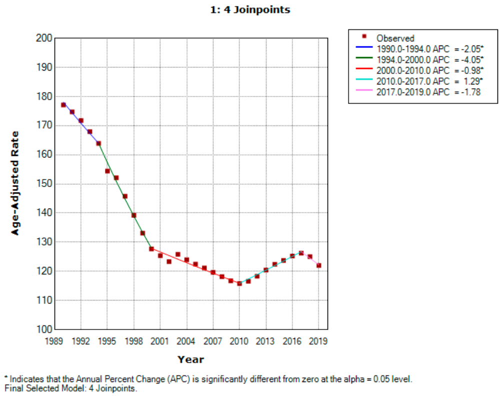
</td>
<td>AAPC = -0.8* (-0.9, -0.7)
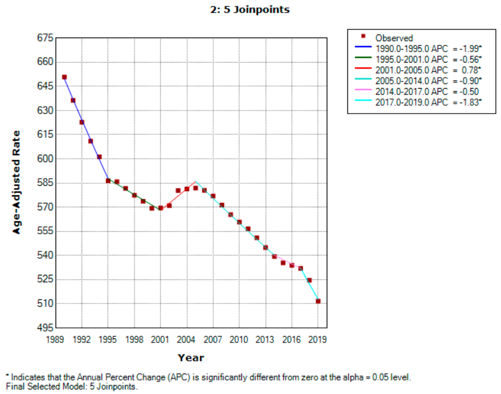
</td>
</tr>
</table>

<table>
<tr><th colspan="4">Upper-Middle income economies ($4046 to $12535)</th></tr>
<tr>
<td>9. Malaysia (0.737)</td>
<td>AAPC = -2.1* (-2.2, -1.9)
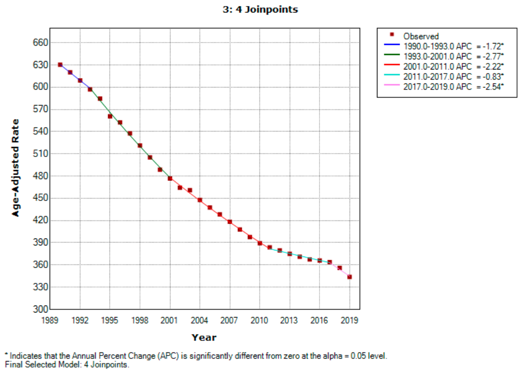
</td>
<td>AAPC = -1.7* (-1.9, -1.5)
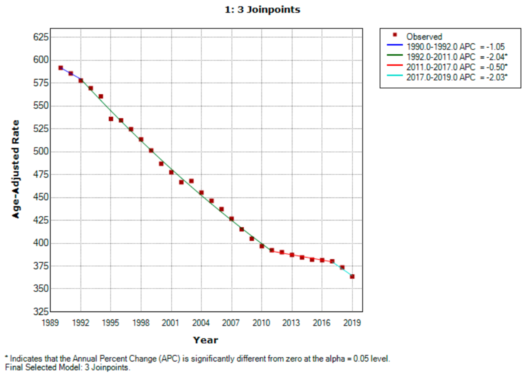
</td>
<td>AAPC = -2.4* (-2.5, -2.3)
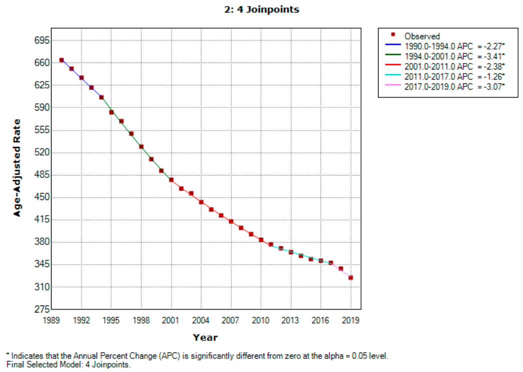
</td>
</tr>
<tr>
<td>10. Niue (0.711)</td>
<td>AAPC = -1.1* (-1.3, -1.0)
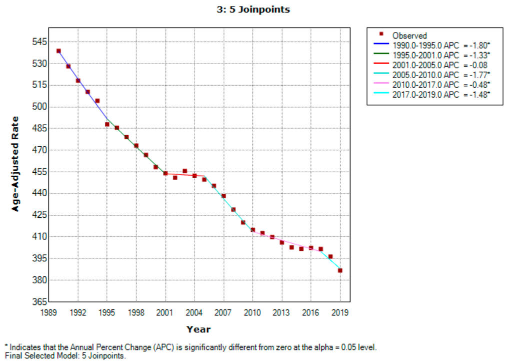
</td>
<td>AAPC = -1.5* (-1.9, -1.1)
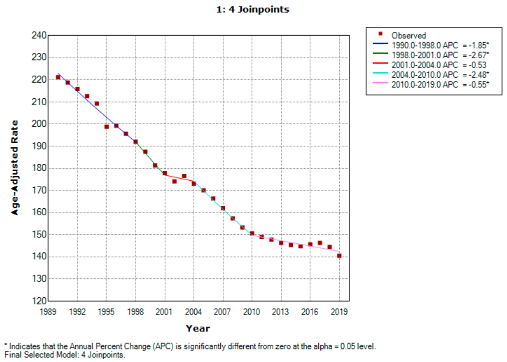
</td>
<td>AAPC = -1.0* (-1.2, -0.8)
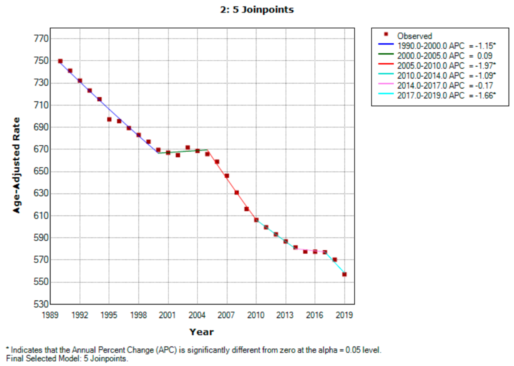
</td>
</tr>
<tr>
<td>11. China (0.686)</td>
<td>AAPC = -3.2* (-3.3, -3.1)
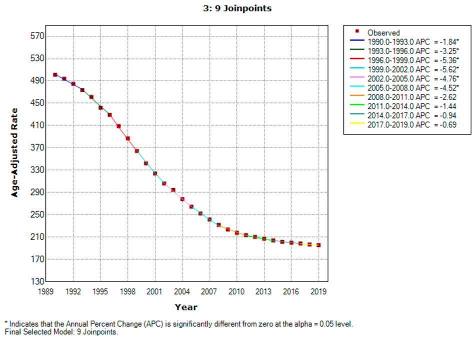
</td>
<td>AAPC = -2.6* (-2.8, -2.4)
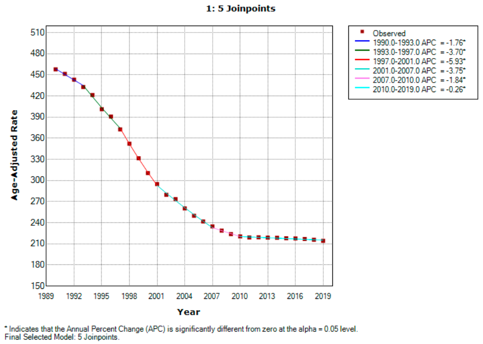
</td>
<td>AAPC = -3.7* (-3.8, -3.6)
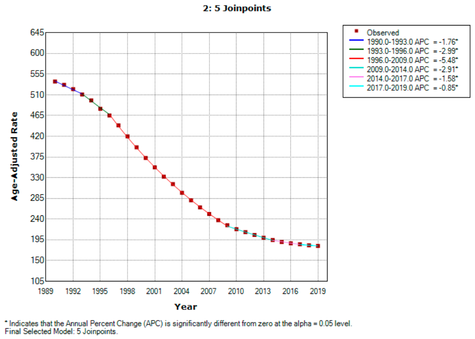
</td>
</tr>
</table>

<table>
<tr>
<td>12. Fiji (0.664)</td>
<td>AAPC = 0.1* (0.0, 0.2)

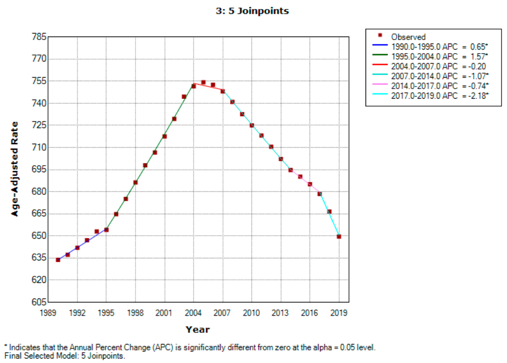
</td>
<td>AAPC = 0.1 (-0.1, 0.2)

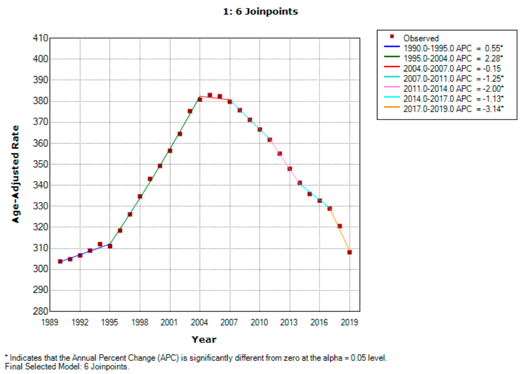
</td>
<td>AAPC = -0.1* (-0.2, -0.1)

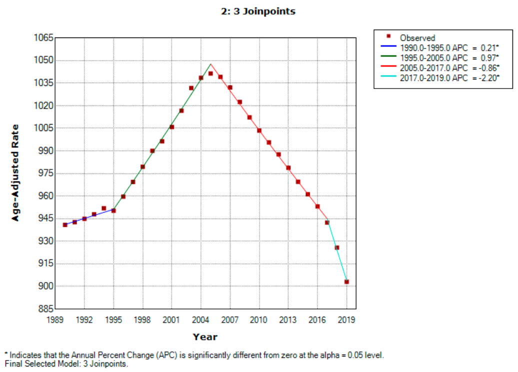
</td>
</tr>
<tr>
<td>13. Samoa (0.641)</td>
<td>AAPC = -0.5* (-0.7, -0.4)

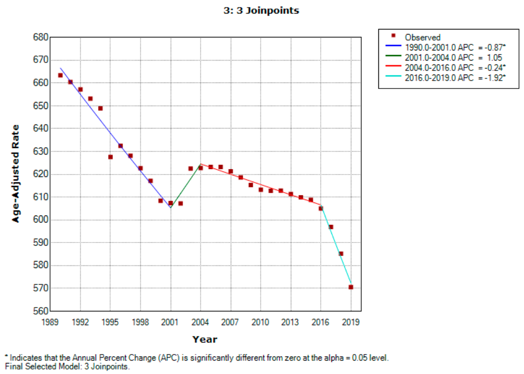
</td>
<td>AAPC = -0.8* (-1.0, -0.6)

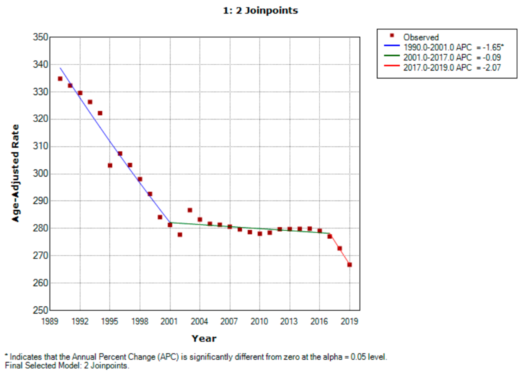
</td>
<td>AAPC = -0.4* (-0.6, -0.3)

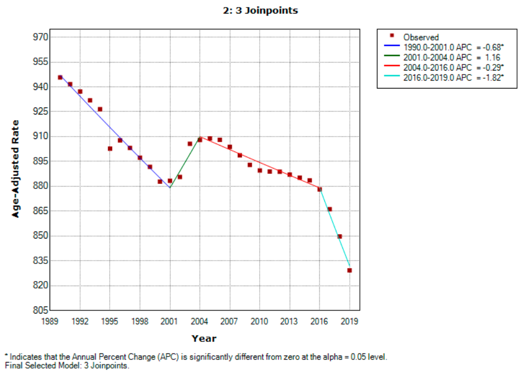
</td>
</tr>
<tr>
<td>14. Tonga (0.636)</td>
<td>AAPC = -0.4* (-0.4, -0.3)

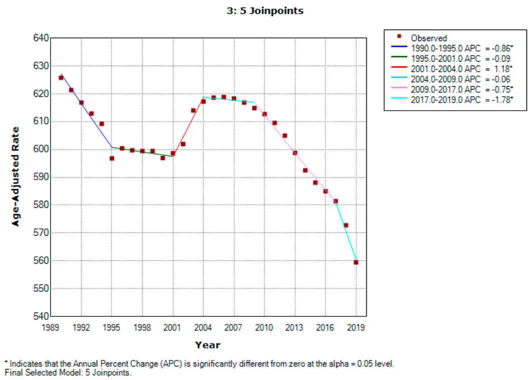
</td>
<td>AAPC = -1.0* (-1.3, -0.7)

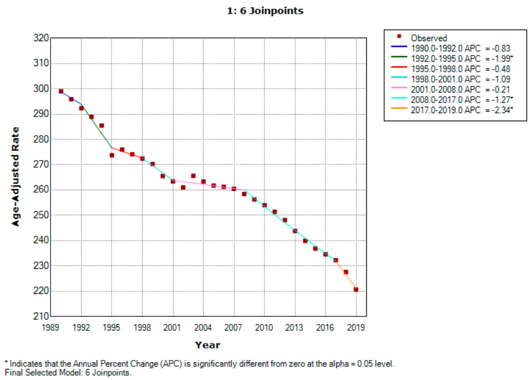
</td>
<td>AAPC = -0.4* (-0.5, -0.3)

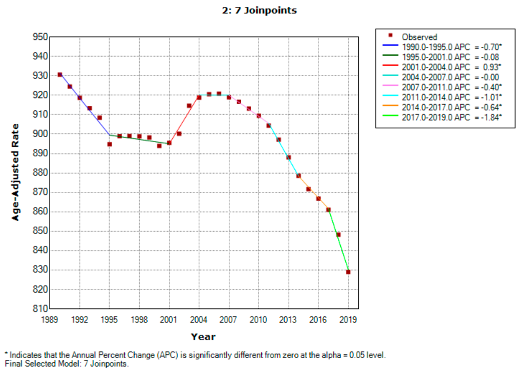
</td>
</tr>
</table>

19. Tuvalu (0.589)

AAPC = -1.0* (-1.1, -1.0)

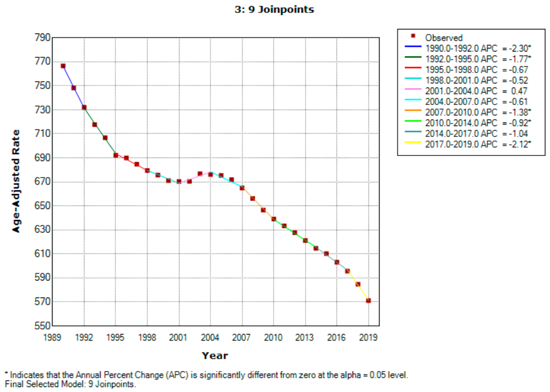

AAPC = -1.5* (-1.6, -1.4)

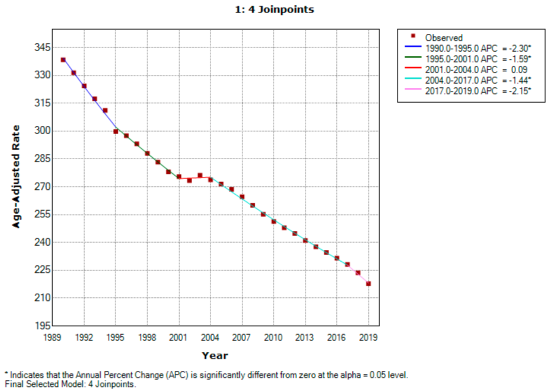

AAPC = -0.7* (-0.8, -0.7)

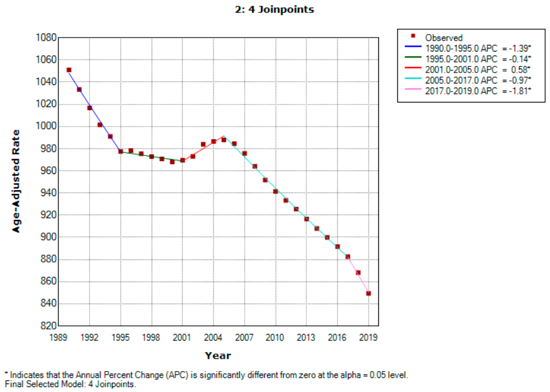

21. Marshall Islands (0.544)

AAPC = -0.8* (-0.9, -0.7)

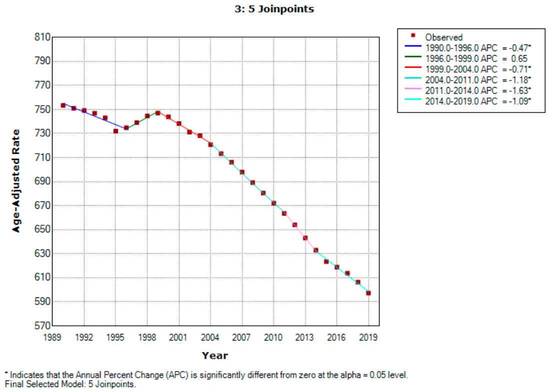

AAPC = =0.9* (-1.0, -0.8)

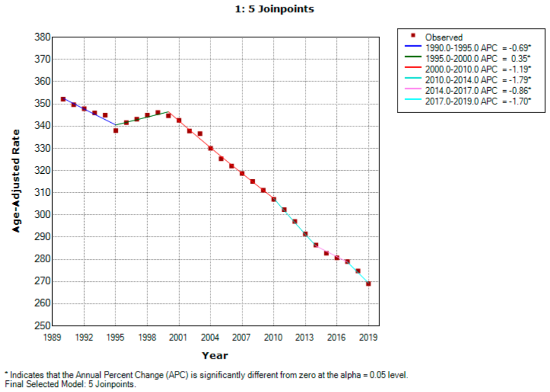

AAPC = -0.4* (-0.4, -0.3)

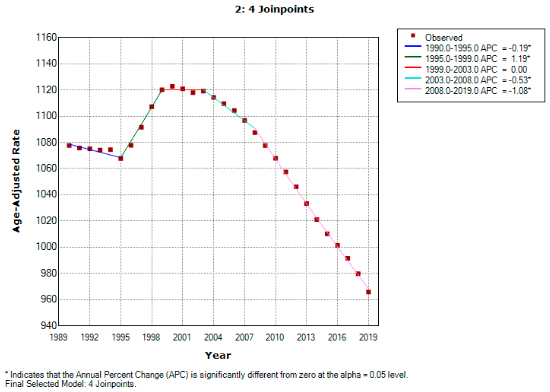

<table>
<tr><th colspan="4">Lower-Middle income economies ($1036 to $4045)</th></tr>
<tr>
<td>15. Philippines (0.623)</td>
<td>AAPC = -1.8* (-1.8, -1.7)
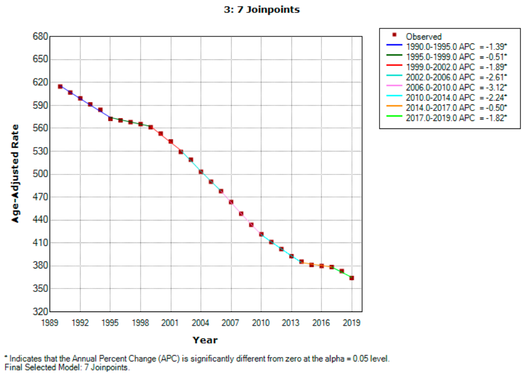
</td>
<td>AAPC = -2.0* (-2.1, -1.9)
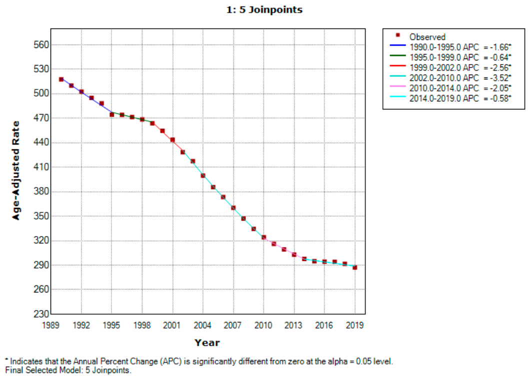
</td>
<td>AAPC = -1.7* (-1.8, -1.7)
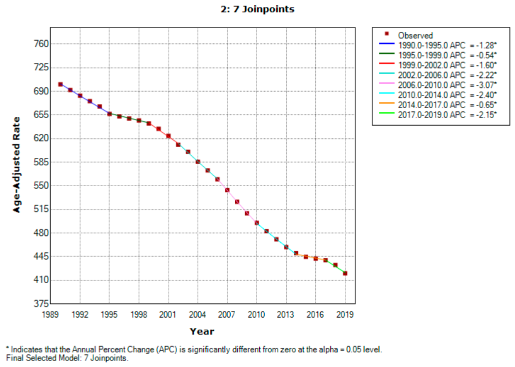
</td>
</tr>
<tr>
<td>16. Nauru (0.618)</td>
<td>AAPC = -0.4* (-0.5, -0.2)
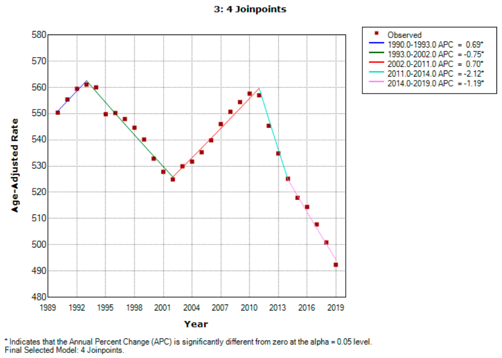
</td>
<td>AAPC = -1.1* (-1.3, -0.9)
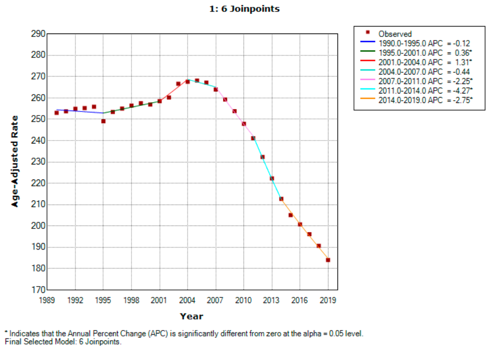
</td>
<td>AAPC = -0.5* (-0.5, -0.4)
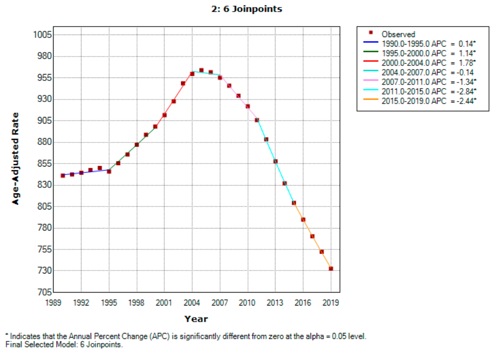
</td>
</tr>
<tr>
<td>17. Vietnam (0.617)</td>
<td>AAPC = -2.6* (-2.7, -2.5)
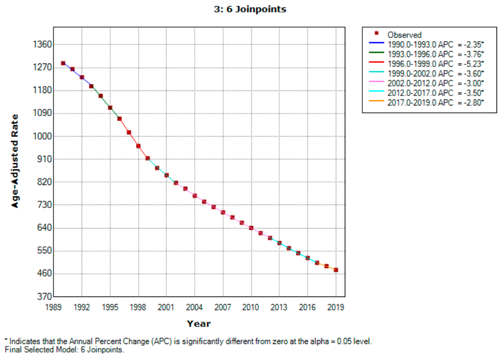
</td>
<td>AAPC = -2.5* (-2.6, -2.3)
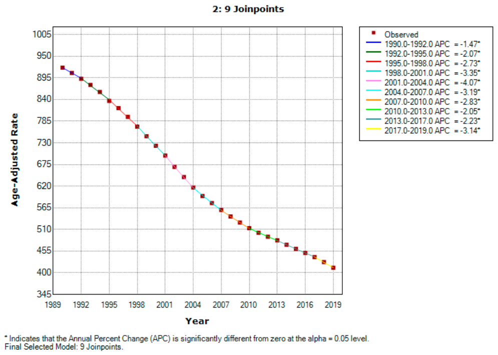
</td>
<td>AAPC = -2.6* (-2.6, -2.6)
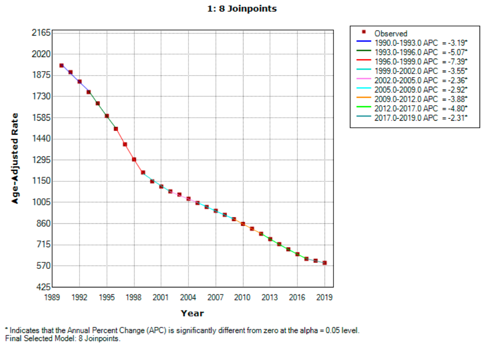
</td>
</tr>
</table>

18. Mongolia (0.606)

AAPC = -1.6* (-1.7, -1.5)

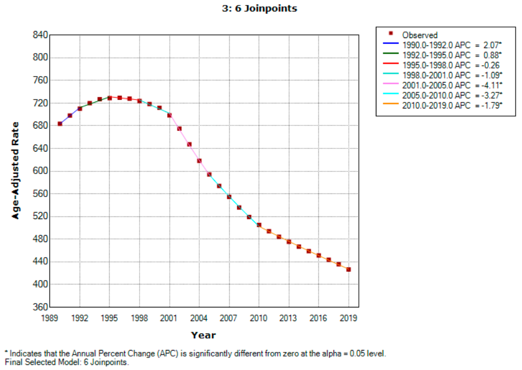

AAPC = -1.7* (-1.7, -1.6)

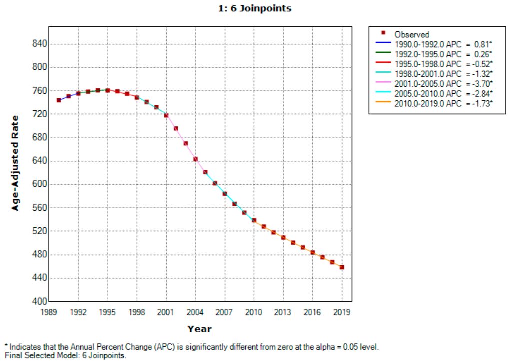

AAPC = -1.6* (-1.7, -1.5)

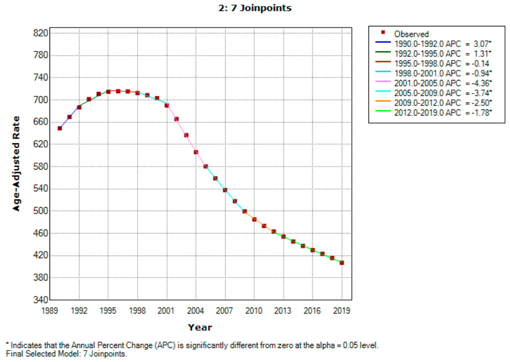

20. Federated States of Micronesia (0.580)

AAPC = -0.7* (-1.0, -0.4)

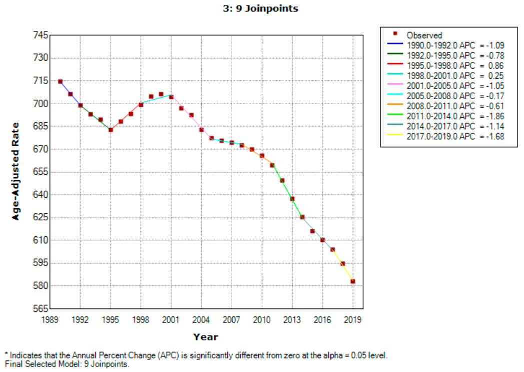

AAPC = -1.5* (-1.7, -1.3)

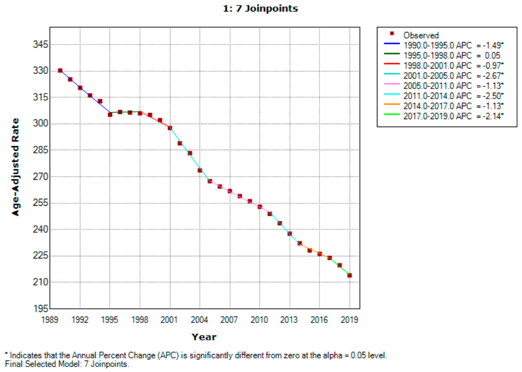

AAPC = -0.7* (-0.7, -0.6)

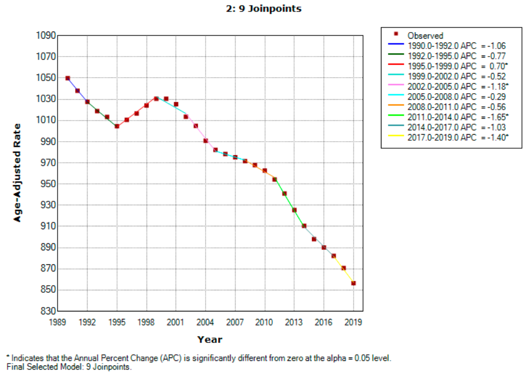

22. Kiribati (0.527)

AAPC = -0.3* (-0.3, -0.2)

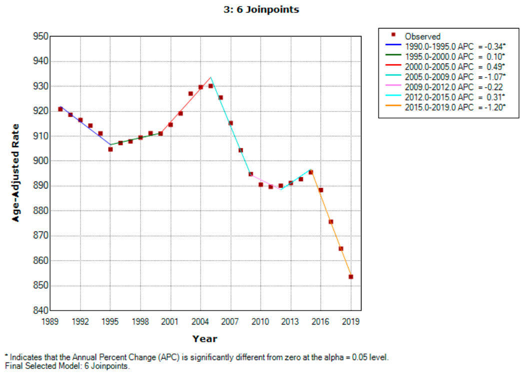

AAPC = -0.9* (-1.0, -0.8)

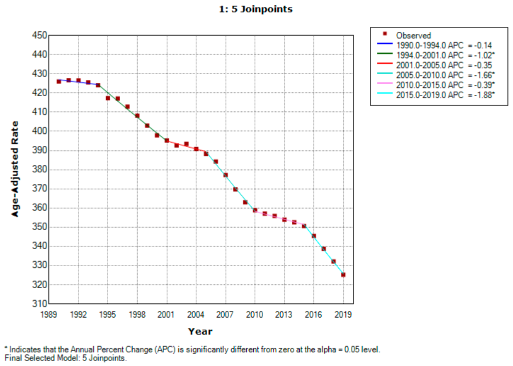

AAPC = -0.2* (-0.3, -0.2)

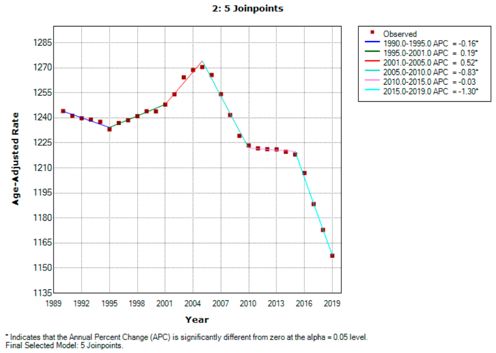

<table>
<tr>
<td>23. Laos (0.49)</td>
<td>AAPC = -1.8* (-1.9, -1.8)
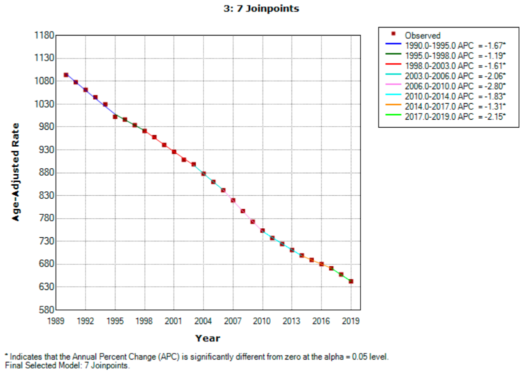
</td>
<td>AAPC = -1.9* (-1.9, -1.8)
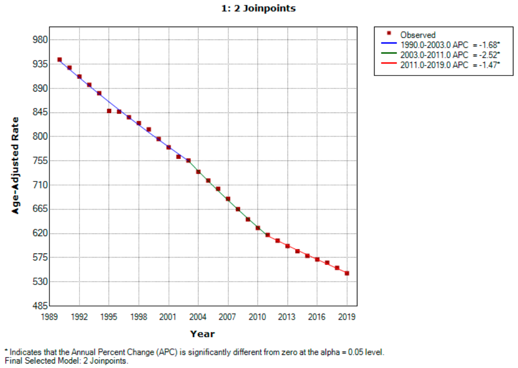
</td>
<td>AAPC = -1.8* (-1.9, -1.7)
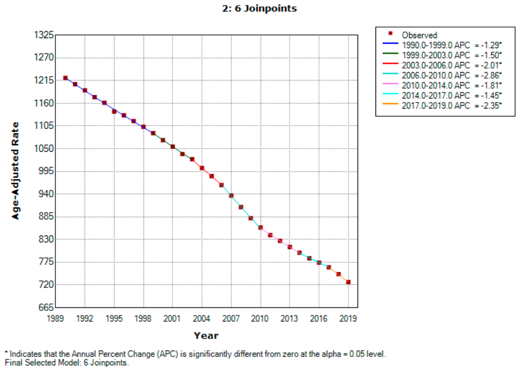
</td>
</tr>
<tr>
<td>24. Vanuatu (0.486)</td>
<td>AAPC = 0.1* (0.0, 0.2)
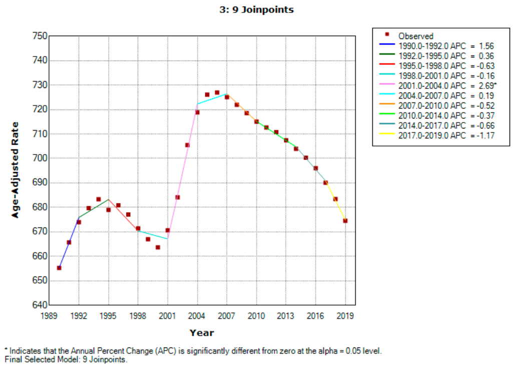
</td>
<td>AAPC = -0.2* (-0.4, -0.0)
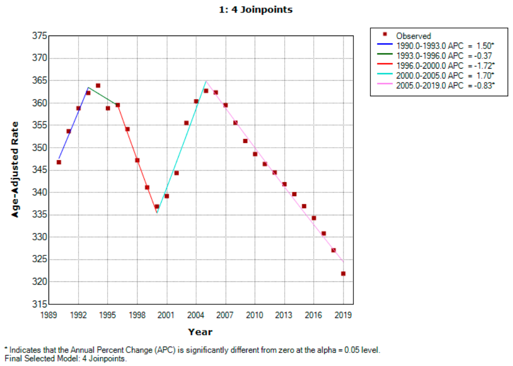
</td>
<td>AAPC = 0.0 (-0.0, 0.1)
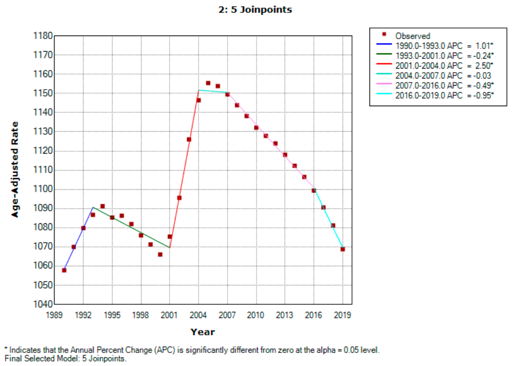
</td>
</tr>
<tr>
<td>25. Cambodia (0.469)</td>
<td>AAPC = -2.0* (-2.1, -1.9)
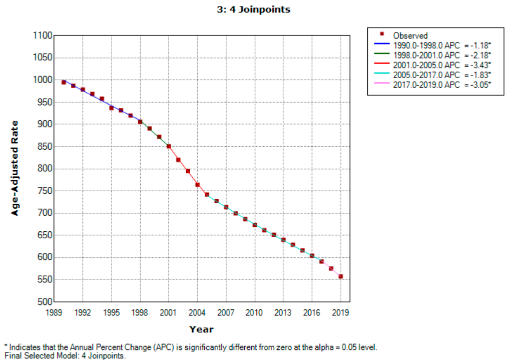
</td>
<td>AAPC = -2.1* (-2.3, -1.9)
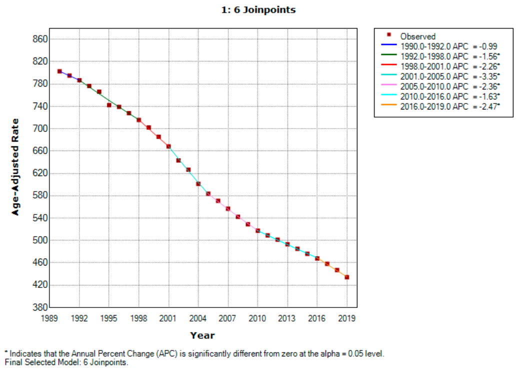
</td>
<td>AAPC = -2.0* (-2.1, -1.9)
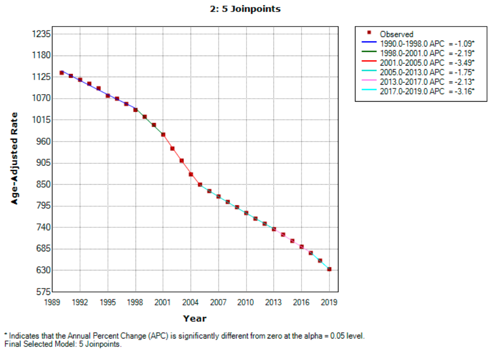
</td>
</tr>
</table>

<table>
<tr>
<td>26. Solomon Islands (0.407)</td>
<td>AAPC = -0.2* (-0.3, -0.1)
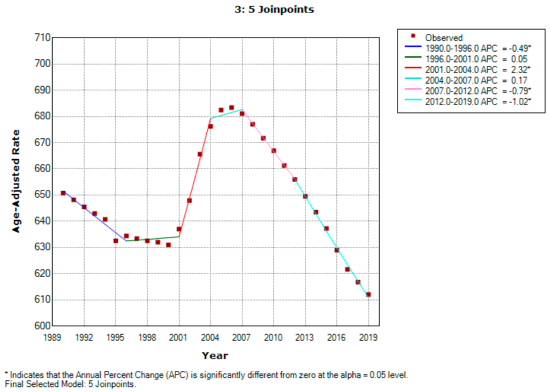
</td>
<td>AAPC = -0.9* (-1.0, -0.8)
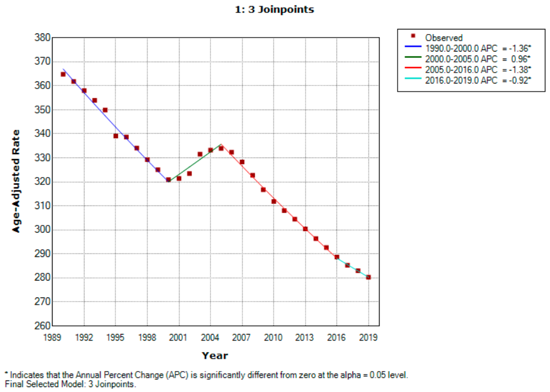
</td>
<td>AAPC = -0.4* (-0.4, -0.3)
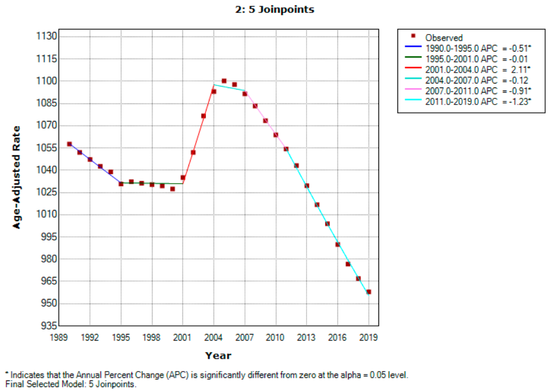
</td>
</tr>
<tr>
<td>27. Papua New Guinea (0.394)</td>
<td>AAPC = -0.3* (-0.4, -0.2)
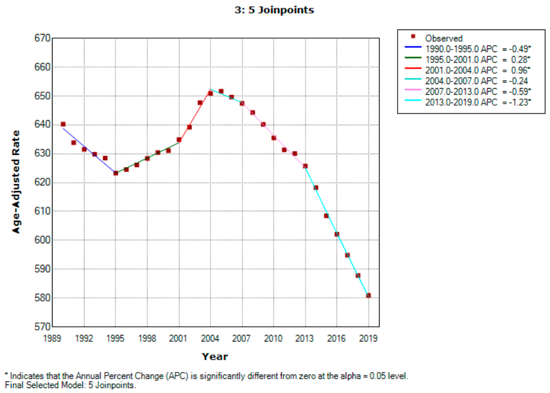
</td>
<td>AAPC = -0.7* (-0.8, -0.6)
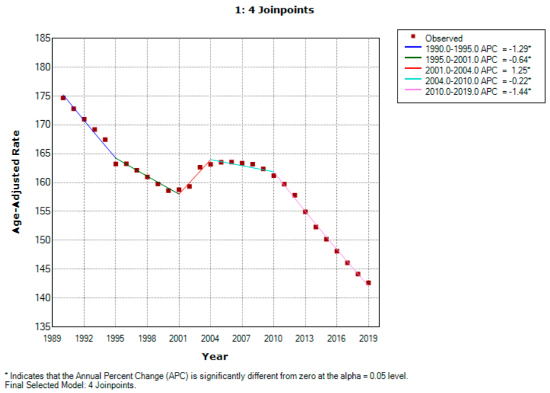
</td>
<td>AAPC = -0.1* (-0.2, -0.1)
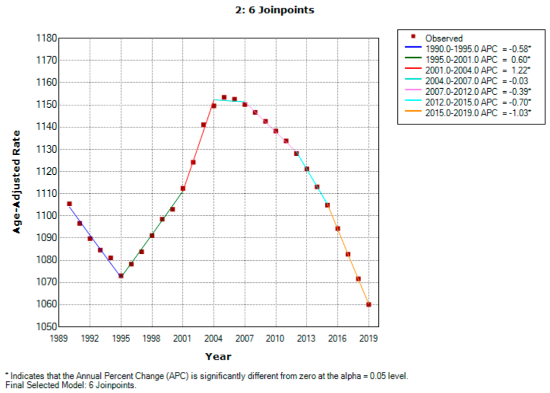
</td>
</tr>
</table>
